# Catalytic Luminescent Polymer Micromotors With Shape‐Programmed Motion

**DOI:** 10.1002/advs.78303

**Published:** 2026-10-22

**Authors:** Yuyang Zhang, Wenyang Huang, Xinwen Gao, Yuhan He, Fei Huang, Tingting Yu

**Affiliations:** ^1^ Shien‐Ming Wu School of Intelligent Engineering South China University of Technology Guangzhou China; ^2^ Institute For Super Robotics (Huangpu) South China University of Technology Guangzhou China; ^3^ Institute of Polymer Optoelectronic Materials and Devices Guangdong Basic Research Center of Excellence for Energy & Information Polymer Materials State Key Laboratory of Luminescent Materials and Devices South China University of Technology Guangzhou China; ^4^ Global Institute of Future Technology Shanghai Jiao Tong University Shanghai China

**Keywords:** blue‐light emission, micromotor/rotor, photocatalytic propulsion, shape programming, spin‐coating strategy

## Abstract

Photoactive polymers hold promise for powering micromotors by converting light into energy. However, traditional energy polymers cannot catalyze on one side of Janus structures in chemically driven micromotors because they only store and convert energy, not perform efficient photocatalysis. In this work, we introduce an indium tin oxide (ITO) conductive interlayer that functions as a dedicated charge‐transfer pathway. This layer efficiently extracts photogenerated holes and spatially separates electron–hole pairs from the PFN‐Br/Pt catalytic layer, thereby creating stable interfacial electric fields and ionic gradients. The combination of self‐electrophoresis and self‐diffusiophoresis enhances propulsion efficiency and long‐term stability compared to traditional single‐catalyst micromotors. The PFN‐Br polymer layer also serves as a fluorescent marker, enabling stable propulsion and real‐time tracking of micromotor movements. Additionally, we show that ultrasonic‐assisted self‐assembly can effectively shape and program these micromotors. This method enables the transition from random movement to more directed trajectories by forming single, dimeric, or trimeric assemblies, which is vital for future investigations of collective dynamics and swarm intelligence. Meanwhile, we have begun initial efforts to utilize its luminescent properties for optical signal encoding, creating opportunities for micro‐ and nano‐scale platforms capable of UV‐controlled optical signaling paired with light‐driven movement.

## Graphical abstract

A Janus micromotor is designed with an SiO_2_/Ti/ITO/PFN‐Br/Pt heterostructure with an engineered energy level. The ITO conductive interlayer facilitates efficient charge separation, generating interfacial electric fields and ionic gradients for enhanced light‐driven propulsion. Ultrasonic‐assisted self‐assembly and fluorescent tracking further enable programmable motion and real‐time visualization, providing a platform for controllable micromotor dynamics and potential optical signaling.

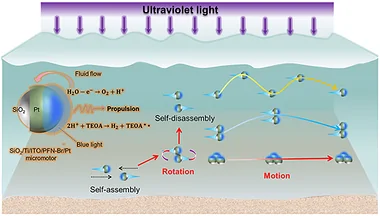

## 1 Introduction

Micro/nanomotors (MNMs) have attracted significant attention in microscale engineering for their ability to convert external energy into controlled locomotion, endowing them with great potential for precision biomedical and environmental applications [1, 2, 3, 4, 5, 6, 7]. Currently, MNMs have been powered by diverse actuation mechanisms, including chemical reactions [8, 9], magnetic actuation [10, 11], sonic excitation [12, 13], and electric stimuli [14]. These external stimuli‐actuated MNMs offer a promising method for on‐demand, sustainable functioning, with fuel‐free, light‐driven propulsion gaining increasing attention due to its wireless, noninvasive nature and precise controllability [15, 16, 17, 18, 19, 20]. Various propulsion mechanisms have been developed for light‐driven MNMs, including optical‐gradient forces, photothermal effects, photocatalytic reactions, and light‐induced mechanical deformation [21]. Chemically light‐driven micromotors mainly operate through two main mechanisms. Photocatalytic propulsion is caused by asymmetric photochemical reactions that generate concentration gradients, and photoelectrochemical reactions produce electric fields, leading to self‐diffusiophoretic and self‐electrophoretic effects, respectively. Due to the complex chemical environment of MNMs, these mechanisms often act simultaneously within the systems, sometimes competing as driving forces in opposite directions [22, 23, 24, 25]. Engineering the motor's chemical system will eventually facilitate the tuning of its two driving mechanisms, potentially influencing the MNM's overall function and movement. However, achieving real‐time tracking, programmable shape, and controllable motion at the same time remains challenging, as these features are typically added as separate modules rather than through a unified design. Consequently, selecting appropriate photoresponsive materials and developing compatible fabrication methods are essential steps for integrating these capabilities into a single light‐driven micromotor.

Currently, research on conventional MNMs primarily uses inorganic semiconductors such as TiO_2_ [26, 27, 28] and BiOI [29] due to their photoelectronic properties, making them crucial materials for constructing light‐driven micro/nanomotor systems. For instance, Zhang et al. [28] designed a simple‐structured micromotor by introducing inherent asymmetry in TiO_2_ crystalline phases. Engineered anatase/rutile TiO_2_ micromotors can achieve efficient self‐propulsion in pure water under UV light through asymmetric surface photocatalytic redox reactions. Dong et al. [29] developed BiOI‐based Janus micromotors that can be driven by a broad spectrum of light, including blue and green, owing to BiOI's outstanding photocatalytic performance. Despite these advances, the inherent rigidity and poor processability of inorganic semiconductors greatly restrict the integration of complex heterostructures within a single micromotor, which in turn limits further functional expansion for practical uses. In this context, developing highly efficient light‐driven MNMs increasingly depends on carefully designed heterostructures that facilitate broad‐spectrum light absorption, effective charge separation and transport, and persistent asymmetric interfacial reactions. Organic semiconductor polymers, with their notable advantages in energy level regulation, molecular design, and interfacial engineering, can effectively satisfy these criteria and are therefore gaining more attention from researchers. Studies show that polymers like Poly[(9,9‐bis(3’‐((N,N‐dimethyl)‐N‐ethylammonium)‐propyl)‐2,7‐fluorene)‐alt‐2,7‐(9,9‐dioctylfluorene)] dibromide (PFN‐Br) are particularly promising due to their effective photoinduced charge separation and inherent blue‐light emission, which enable both controllable propulsion and real‐time response visualization [30, 31]. Therefore, this study proposes a viable engineering strategy for the integrated assembly of multiple functional modules on a single micromotor using the PFN‐Br, offering a scalable solution to address challenges in the design of multifunctional light‐driven micro‐nano systems. Specifically, PFN‐Br acts as the photoactive semiconductor and fluorescent marker, while Pt offers the reduction‐active interface. ITO is added as a conductive interlayer to facilitate directional charge transport. This multilayer structure supports effective photoinduced charge separation and movement, enabling programmable motion within a single micromotor.

In this work, multifunctional heterostructure micromotors (SiO_2_/Ti/ITO/PFN‐Br/Pt) are fabricated by combining electron‐beam evaporation and a spin‐coating‐assisted process (Figure 1a). To achieve efficient photoresponse, an energy‐level gradient across PFN‐Br, ITO, and Pt is engineered through a multilayer sandwich‐like structure. As illustrated in Figure 1a, the energy diagram shows that upon UV excitation, photogenerated electrons in PFN‐Br populate the LUMO and are injected directionally into the Pt layer, where they participate in the hydrogen evolution reaction (HER) [31]. Meanwhile, photogenerated holes migrate toward the ITO layer [32], where oxidative reactions take place. This bidirectional charge transfer establishes stable ionic gradients and interfacial electric fields, forming the basis for self‐electrophoretic and self‐diffusiophoretic propulsion. Consequently, the micromotors exhibit competitive propulsion capabilities comparable to typical photocatalytic micro/nanorobotic systems [17, 18, 33, 34, 35, 36, 37, 38, 39, 40, 41, 42] (Figure 1b and Table S1). In addition to propulsion, the present system is designed to enable programmable regulation of micromotor shape. Unlike methods that use ultrasonication solely to disperse micromotor particles, this study applies ultrasonic processing to achieve shape programming. Adjusting ultrasonic parameters allows the micromotor to self‐assemble into various configurations, such as single (SL), dimers (DE), and trimers (TR‐I/TR‐II) (Figure 1a). These shape‐programming modifications alter the torque balance and limit movement freedom, leading to improved control over the micromotor's motion. This makes the micromotors more likely to move in a directed, linear manner rather than randomly. Simultaneously, adding the PFN‐Br layer imparts inherent optical properties, with bright blue emission that enables real‐time tracking of micromotor paths under optical microscopy (Figure S1) and offers a means for optically encoded signal feedback during movement. Unlike traditional light‐driven micromotors that focus solely on propulsion, this energy‐level‐engineered ITO/PFN‐Br/Pt heterostructure combines directed photoinduced charge movement with shape‐dependent motion control and fluorescence‐based tracking within a single micromotor. Instead of viewing propulsion, movement regulation, and fluorescence as separate functions, this integrated system creates a multifunctional platform that links controlled movement with externally controllable optical signals. This approach lays the foundation for more sophisticated, interactive micromotor systems.

**FIGURE 1:**
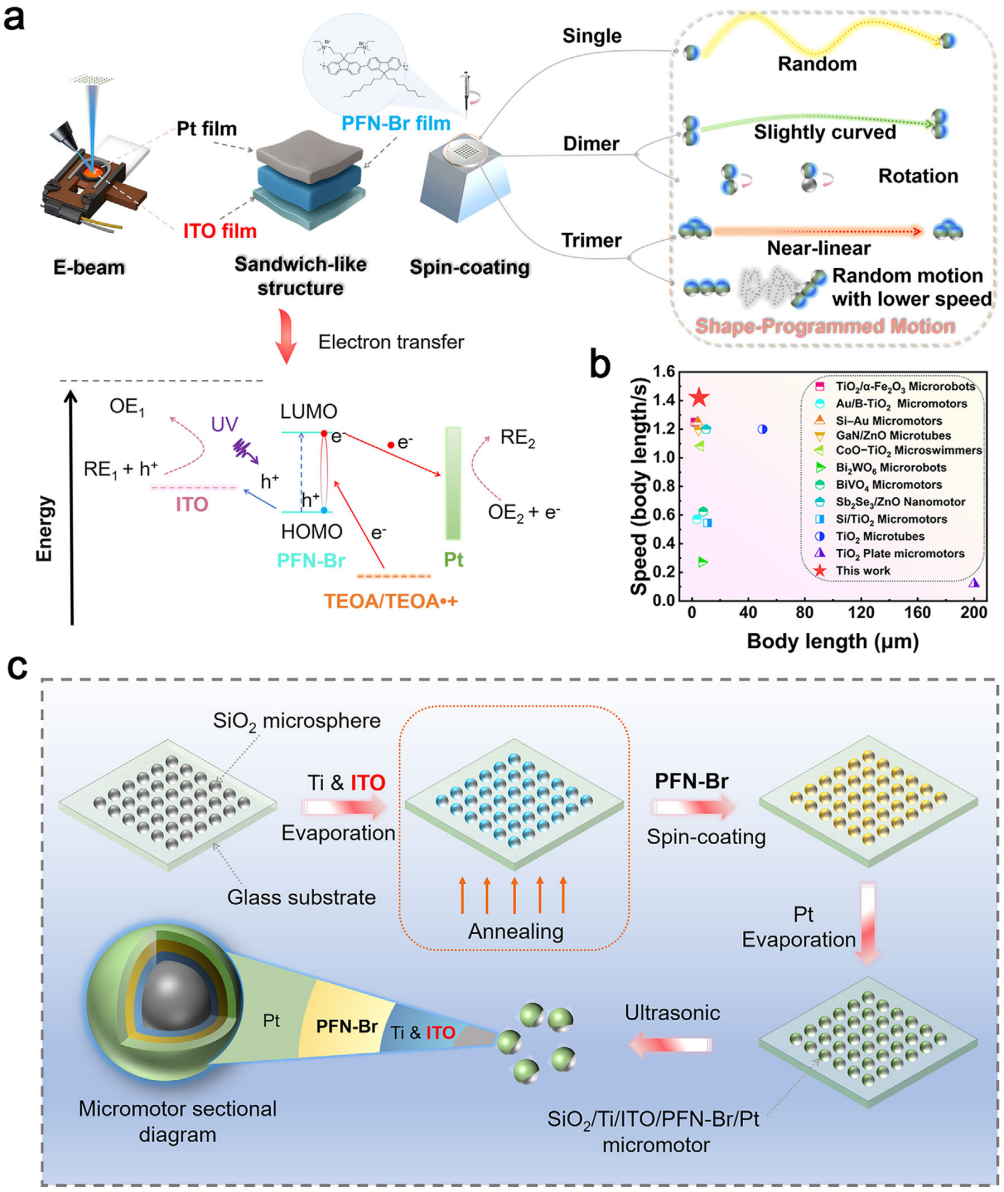
(a) Multi‐configuration UV‐driven micromotor fabricated based on E‐beam evaporation, combined with a modular spin‐coating‐assisted assembly process, and its approximate energy band diagram. (b) Comparison of some reported micromotors plotted as the ratio of locomotion speed to body length as a function of length. (c) Schematic illustration of the fabrication process.

## 2 Results and Discussion

### 2.1 Design and Fabrication of the SiO_2_/Ti/ITO/PFN‐Br/Pt Micromotors

In this study, Janus spherical micromotors were produced using the process shown in Figure 1c. SiO_2_ microspheres on the substrate were first coated with a Ti layer by electron‐beam evaporation to serve as an adhesive. To optimize the electronic properties, an ITO layer was then deposited on the microspheres. High‐temperature annealing converted this into a transparent film, confirming the successful formation of the ITO layer. Next, the organic semiconductor PFN‐Br was spin‐coated onto the surface. A Pt layer was then evaporated onto the microspheres to serve as a catalyst, yielding SiO_2_/Ti/ITO/PFN‐Br/Pt micromotors. Finally, ultrasonication released these microspheres into pure water, demonstrating a reliable, large‐scale method for fabricating micromotors.

As depicted in Figure 2a, scanning electron microscopy (SEM) images show that the micromotors are evenly dispersed and display a dense, spherical shape on the substrate, with an average size of around 5 µm. High‐resolution SEM images of a single microsphere (Figure 2b) demonstrate successful integration of multilayer functionalized films, forming a Janus micromotor. Additionally, energy‐dispersive X‐ray spectroscopy (EDS) mapping confirms the elemental distribution: Br, Sn, In, and Pt are mainly concentrated at the bottom of the micromotor, whereas Si and O are uniformly distributed across the surface. Corresponding spectral details are provided in Figure S2. Further, X‐ray diffraction (XRD) analysis confirms the development of the multifunctional layered structure (ITO, PFN‐Br, and Pt film), as shown in Figure S3. More detailed analysis is available in the Supporting Information. The surface topography and thickness of the PFN‐Br layer were characterized using a surface profilometer (Figure 2c and Figure S4). Step‐height measurements at five different positions across the PFN‐Br to glass interface indicated an average film thickness of around 197.5 nm, aligning well with the 3D topographic reconstruction. As shown in Figure S5, the micromotor spectrum exhibits a broader absorption range than that of individual PFN‐Br films, with notable peaks at 335 and 426 nm. These peaks result from molecular aggregation and excitonic coupling in the multilayer film heterostructure [43, 44]. The expanded photoresponsive range enhances the micromotor's activity under light. The inset in Figure S5 shows an image of real micromotors, illustrating a quick method for producing scalable micromotors. Additionally, when exposed to 365 nm UV light, the sample glows blue (see the insert in Figure S6), and its fluorescence spectrum (Figure S6) reveals two peaks at 422 and 441 nm. This is due to PFN‐Br's natural luminescence mechanism, involving the radiative recombination of electron–hole pairs material [31, 38]. This fluorescence can be rapidly quenched and restored by switching the UV light on and off, a property that offers significant potential for real‐time detection of micromotor motion.

**FIGURE 2:**
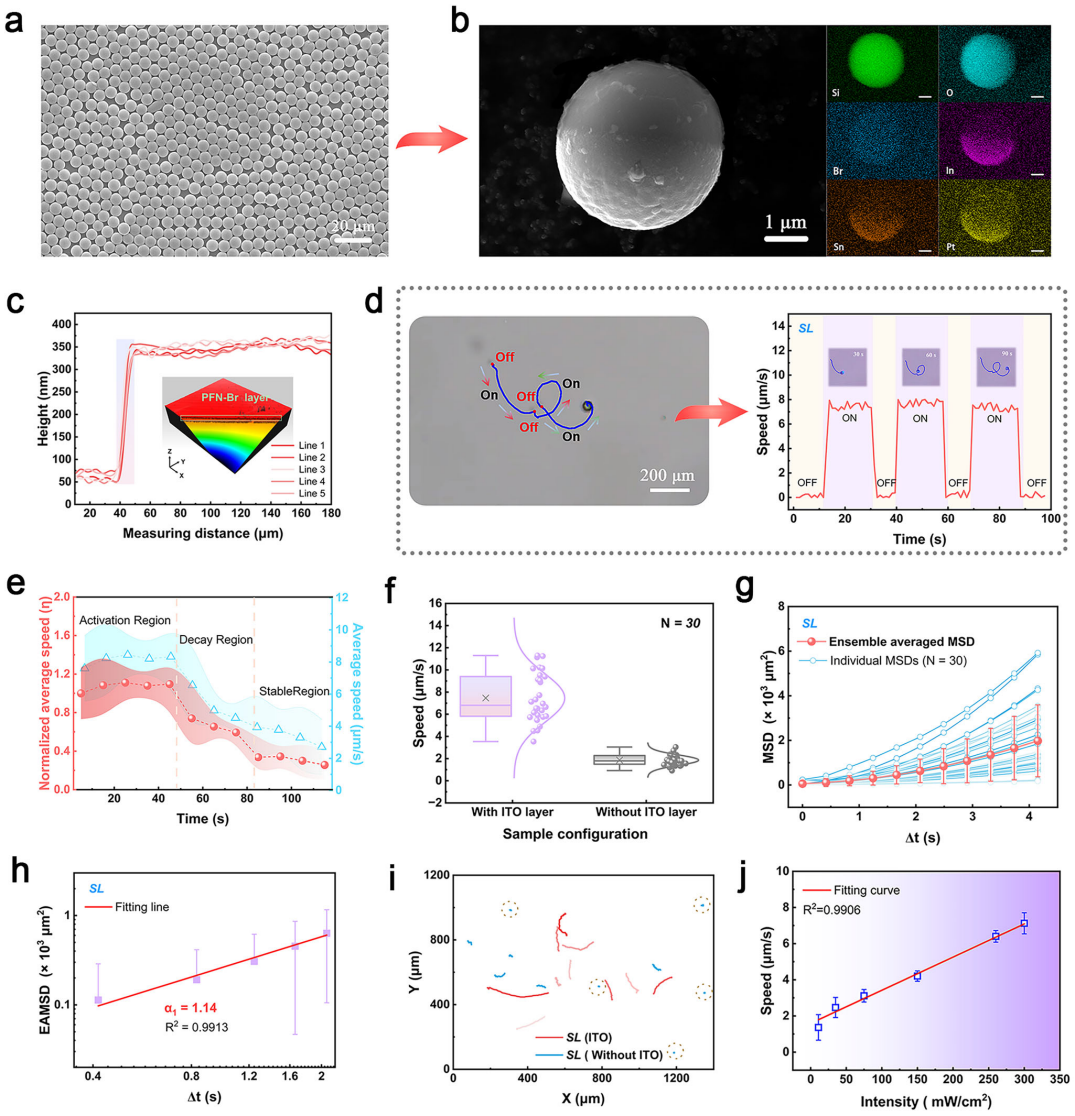
Characterization of the SiO_2_/Ti/ITO/PFN‐Br/Pt micromotor. (a) SEM image of a dense array of micromotors on a substrate. (b) High‐magnification SEM image of an individual micromotor with corresponding EDS elemental mappings for Si, O, Br, In, Sn, and Pt. (c) Line profiles of measured lines for layer thickness analysis; 3D stereoscopic topography of the PFN‐Br layer (inset graph). (d) Trajectory and instantaneous speed of the micromotor under UV light (300 mW cm^−^^2^) over 90 s. (e) The normalized average speed analysis of the micromotor under UV light. (f) Average speeds of micromotors with and without an ITO layer. Each data point corresponds to an individual micromotor (N = 30 per group), with the mean speed derived from a 20 s trajectory. Box plots illustrate the median, interquartile range, and whiskers, alongside the distribution of individual data points. (g) The mean squared displacement (MSD) curves for 30 micromotors and the ensemble‐averaged MSD (EAMSD) over lag time (Δt). Blue gradient lines depict individual MSDs, while the red line indicates the EAMSD from all trajectories. Error bars show the standard deviation. (h) A log‐log plot of EAMSD vs. lag time (Δt) based on 30 trajectories of 20 s each, with a power‐law fit over the first 2 s (red line). Error bars represent the standard deviation. (i) Typical trajectories of selected micromotors with and without an ITO layer (n = 10 per group). (j) Average micromotor speed relative to light intensity, with each point representing the mean speed from 15 micromotors. Error bars indicate standard deviation.

### 2.2 Behavior Analysis of Micromotor Motion

The organic semiconductor PFN‐Br layer's ability to emit blue light from incident photons enables real‐time detection and efficient locomotion even in low‐light conditions. As shown in Figure S7 and Movie S1, under UV irradiation, the SiO_2_/Ti/ITO/PFN‐Br/Pt micromotor exhibited a relatively low movement speed of approximately 1.64 µm s^−^^1^ in pure water. The addition of TEOA as a sacrificial hole scavenger significantly improved the propulsion by suppressing photogenerated electron–hole recombination and enhancing interfacial redox reactions [31]. The optimal TEOA/H_2_O ratio for micromotor propulsion was found to be 1/9 (see Supporting Information for details). As shown in Figure 2d and Movie S2, the light‐driven propulsion of the ITO‐containing SiO_2_/Ti/ITO/PFN‐Br/Pt micromotors was tested under intermittent UV light in TEOA/H_2_O solution.

Without UV exposure, the micromotors exhibited only Brownian motion with minimal displacement. When UV light was applied, the photoelectrochemical (PEC) process was initiated, leading to directional movement and the emission of the characteristic blue fluorescence of PFN‐Br [30]. At a UV intensity of 300 mW cm^−^^2^, the micromotors reached an average propulsion speed of approximately 7.13 µm s^−^^1^. Repeated cycles of turning UV on and off allowed for quick, reversible switching between moving and stationary states, confirming light‐controlled reversible propulsion. The stability of this motion was examined throughout the experiment. During UV exposure, the ITO‐containing micromotors showed a typical three‐phase pattern: activation, decay, and a subsequent stable phase (Figure 2e). The average speed was measured in 10 s intervals, using the initial 10 s's speed (7.50 µm s^−^^1^) as a reference for normalization (η = s_t_/s_ref_, details see the Methods section in the Supporting Information). As illustrated in Figure 2e, during the activation region (0–50 s), the normalized average speed (η) remained stable at around 1.0, reaching a maximum of 1.11 with an average speed of 8.16 µm s^−^^1^. This shows that the micromotor exhibited promising speed and stable motion within this region. Later, during the decay phase (50–80 s), a decrease in propulsion was observed, with both normalized and average speeds gradually dropping. After 80 s, the micromotors stabilized at a lower, steady propulsion speed, rather than stopping completely. By the end of the 120 s observation period, η was still at 0.31, indicating that the micromotors kept roughly one‐third of their initial propulsion ability. Narrow error bands indicate consistent and repeatable motion. This decay process is similar to that observed in previous research [2]. In contrast, the ITO‐free SiO_2_/Ti/PFN‐Br/Pt micromotors mainly moved randomly during the 90 s observation. Their fluctuating speeds showed no clear link to the cyclic UV on/off switching (see Movie S2 and Figure S8). Therefore, although they had nonzero speeds due to random motion, they did not demonstrate continuous photoactivated propulsion.

Analysis of 30 micromotors revealed a notable decline in propulsion efficiency after the ITO layer was removed. Micromotors with ITO averaged a speed of about 7.47 µm s^−^^1^, while the ITO‐free controls moved much slower at only 1.85 µm s^−^^1^, mostly due to random motion (Figure 2f). This was quantified through mean square displacement (MSD) analysis. The ITO‐equipped micromotors exhibited significantly higher MSD values as lag time increased (Figure 2g), whereas the ensemble‐averaged MSD (EAMSD) of the ITO‐free samples remained much lower (Figure S9a). Power‐law fitting based on EAMSD∝Δt^α^ yielded an exponent of α_1_ = 1.14 for the ITO‐containing micromotors, indicating a superdiffusive behavior linked to active propulsion [45, 46]. Removing the ITO layer reduced the exponent to α_2_ = 0.81, suggesting primarily subdiffusive random motion without consistent effective displacement (Figures 2h and S9b). Similarly, analysis of locomotion trajectories revealed that micromotors with ITO traveled considerably farther and exhibited more directed movement compared to the controls without ITO (Figure 2i). In the PCE process, the photon flux is directly related to the incident photocurrent intensity [47]. Meanwhile, the electrophoretic driving force mainly depends on the self‐generated electric field strength, which reflects the magnitude of the photo‐induced current. It is directly proportional to the light intensity [48]. As shown in Figure 2j, increasing light intensity enhances the SiO_2_/Ti/ITO/PFN‐Br/Pt micromotors’ motion speed (Movie S3), consistent with the Hückel framework, which states that a particle's electrophoretic speed depends on the inter‐electric field [49]. A linear correlation exists between light intensity and motion speed, indicating that motion speed can be controlled via light modulation. This supports the proposed self‐electrophoresis propulsion mechanism driving the micromotor's movement. The mean squared displacement (MSD) further confirmed the light‐driven propulsion effect (Figure S10). As the light intensity varies, a series of nonlinear fitting curves was obtained for the light‐driven propulsion of micromotors from 35 to 260 mW cm^−2^, confirming light power‐dependent motion similar to typical photocatalytic MNMs (see the Supporting Information for more detailed analysis) [50].

### 2.3 Propulsion Mechanism of Micromotors

The proposed propulsion mechanism of the SiO_2_/Ti/ITO/PFN‐Br/Pt single micromotor is illustrated in Figure 3a. Its motion mainly results from self‐electrophoresis, which significantly drives propulsion under UV irradiation. Additionally, reaction‐induced chemical gradients can also contribute via self‐diffusiophoresis. A multilayer film heterostructure has been constructed on the micromotor, with PFN‐Br deposited onto the ITO conductive layer, followed by a Pt catalyst layer, forming a “sandwich‐like” structure that promotes efficient charge separation and transfer during reactions, explaining its effective movement under UV light. When illuminated, the PFN‐Br layer absorbs photons, generating electron–hole pairs. This excitation prompts an electron transition from the highest occupied molecular orbital (HOMO) level to the lowest unoccupied molecular orbital (LUMO) level of PFN‐Br, and subsequently participates in the Pt catalytic reaction. To accelerate this process, the engineering of energy levels is indispensable. The ITO (≈ −4.7 eV) is a promising construction candidate owing to its well‐aligned energy levels with PFN‐Br (HOMO ≈ −5.7 eV), which enable efficient hole extraction and enrichment in the active hemisphere. The energy level values have been confirmed by relevant reports [31, 32]. Meanwhile, triethanolamine (TEOA) acts as a sacrificial hole scavenger, preventing electron–hole recombination (Equation 1) and thereby ensuring continuous charge transfer [51], which sustains PEC processes. To directly detect the reactive intermediates formed during photoexcitation, electron paramagnetic resonance (EPR) spectroscopy was used with DMPO as a spin‐trapping agent (see Figure 3b). Under UV light, a clear DMPO‐•O_2_^−^ signal appears, while this signal is almost absent in the dark, offering direct spectroscopic proof of superoxide radical production upon illumination. Additionally, a much weaker DMPO‐•OH signal is observed under light conditions. These findings confirm that transient reactive oxygen species are produced upon photoexcitation, supporting the occurrence of photoinduced interfacial redox reactions within the micromotor system [23, 52]. The effect of the ITO interlayer on the photoluminescence (PL) of the micromotors was further analyzed under 365 nm excitation (see Figure 3c). Both the ITO‐present and ITO‐absent micromotors showed the typical blue emission of PFN‐Br at around 422 and 441 nm. Importantly, the ITO‐free micromotors displayed an extra emission peak near 532 nm, which was significantly reduced when the ITO interlayer was added. This suggests that the ITO layer greatly influences the excited‐state relaxation processes and interfacial carrier dynamics in PFN‐Br [53]. Combined with the favorable energy‐level alignment for hole transfer from PFN‐Br to ITO, this spectrum change aligns with modified interfacial charge‐transfer processes. It corroborates the proposed function of ITO in promoting efficient hole extraction and transport. Both the EPR and PL results support a photoinduced process that involves interfacial carrier transfer followed by redox reactions within the heterostructured micromotor.

**FIGURE 3:**
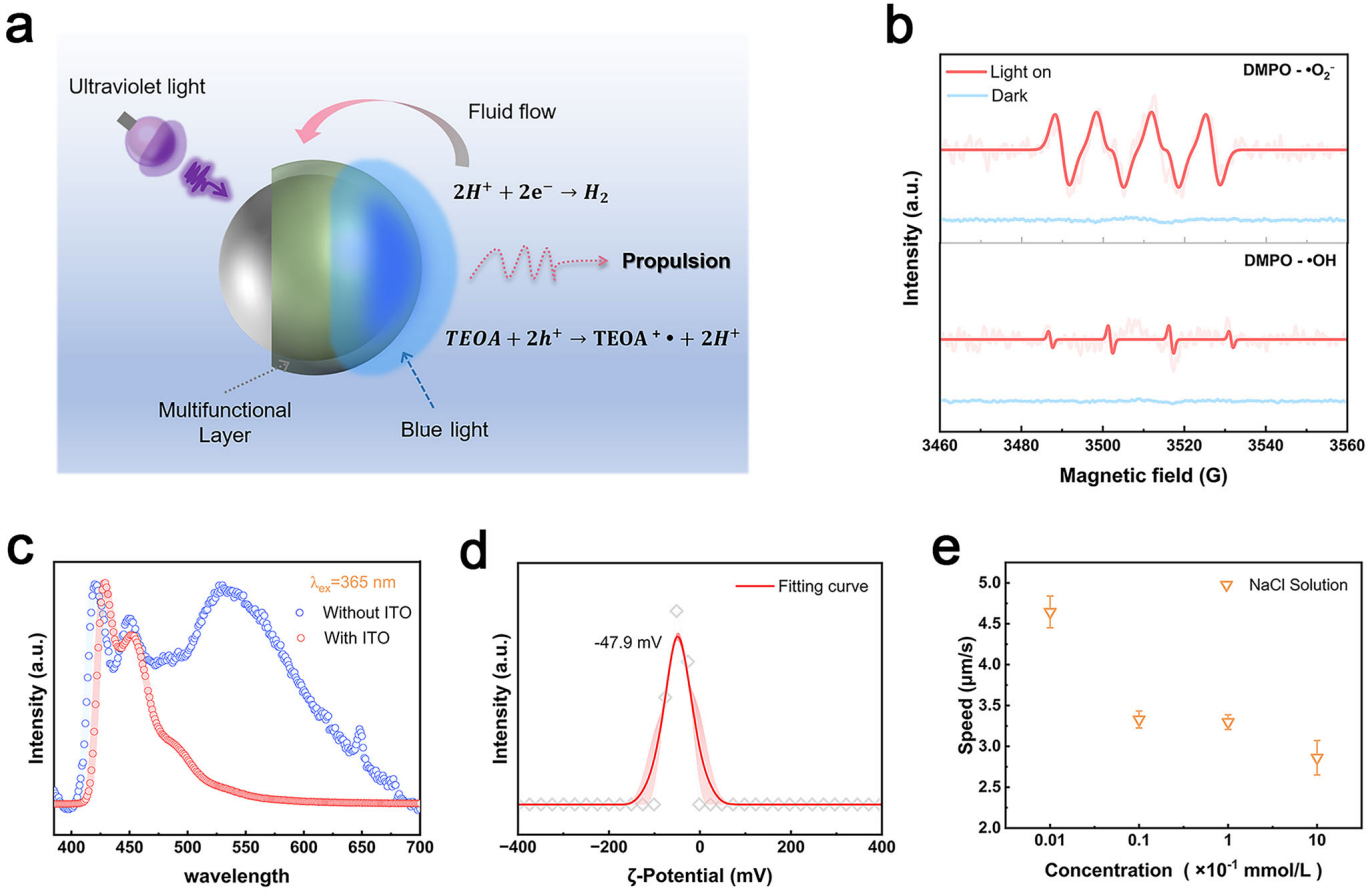
The driving mechanism of the SiO_2_/Ti/ITO/PFN‐Br/Pt micromotor. (a) Schematic of the UV‐induced propulsion mechanism. (b) EPR spectra of DMPO‐•O_2_^−^ and DMPO‐•OH adducts generated by micromotors upon UV irradiation for 5 min. (c) Fluorescence emission spectrum of the micromotor (With and without ITO layer). The excitation wavelength λ_*ex*_ is 365 nm. (d) Zeta potential of pristine silica microspheres. (e) Speed of the micromotor as a function of NaCl concentration. Error bars represent standard deviation.

Due to the structural and chemical asymmetry of the Janus micromotor, photoinduced reactions occurring in different regions are likely to cause uneven distributions of ionic and molecular species in the surrounding solution. According to electrokinetic theories of self‐electrophoretic micromotors, these asymmetric surface reactions produce heterogeneous ionic fluxes, resulting in variations in electric potential and an internally generated electric field that drives propulsion [54, 55]. The negative zeta potential observed (Figure 3d) confirms a charged interface between the micromotor and the solution, facilitating electrokinetic interactions with surrounding ions. However, it does not specify the magnitude or spatial arrangement of the self‐generated electric field. This is consistent with the behavior of self‐electrophoretic micromotors, where uneven surface reactions produce nonuniform ionic distributions and local electric fields that drive propulsion [55]. Based on the self‐electrophoretic propulsion model, the net surface charge field drives ionic flow from the active hemisphere toward the inactive hemisphere, thereby propelling the micromotor along the dipole axis toward the Pt‐coated side. Since the active hemisphere of micromotors emits blue light during operation, the direction of this motion can be easily verified experimentally (Movie S2), further supporting the proposed self‐electrophoresis mechanism.

In parallel, the PFN‐Br strategy for hydrogen production with UV illumination with Pt assistance has been demonstrated to be feasible [31]. The active hemisphere of micromotors promotes the hydrogen evolution reaction (HER, Equation 2) when exposed to UV‐light. In this process, the consumption of H^+^ ions may locally alter the ionic environment near the Pt surface. Rather than implying a directly measured pH gradient, this effect is more appropriately understood as a potential source of local variations in ion concentration. Because of the asymmetric Janus structure, the uneven consumption of reaction species may create local concentration gradients, potentially enabling an extra self‐diffusiophoretic effect. This is consistent with the EPR measurements. Consequently, the motion of the micromotors indicates a combined propulsion mechanism that includes both substantial self‐electrophoresis and additional self‐diffusiophoresis.

(1) $$TEOA+2h^{+}\to \mathrm{TEO}A^{+}•+2H^{+}$$

(2) $$2H^{+}+2e^{-}\to H_{2}$$

To better understand the motion mechanism of this micromotor, its average speed was measured at different NaCl concentrations, as shown in Figure 3e. The role of self‐electrophoresis is further supported by how propulsion depends on ionic strength. When NaCl concentration rises from 0 to 0.1 mmol L^−^^1^, the average speed drops by about 2.2 times, from 7.13 to 3.20 µm s^−^^1^. Higher ionic strength increases electrostatic screening and compresses the electric double layer, which diminishes the effectiveness of the self‐generated electric fields in driving electrokinetic motion [55, 56, 57, 58]. The clear salt‐related reduction in propulsion aligns with a self‐electrophoretic effect. Notably, the speed (2.75 µm s^−^^1^) remains above zero even at higher ionic strength, indicating that photocatalytic hydrogen evolution on the Pt surface continues to offer an extra self‐diffusive driving force [48], allowing the micromotor to retain limited motility under such conditions. Importantly, self‐electrophoretic and self‐diffusiophoretic effects should not be regarded as completely independent in chemically active micromotors. As shown in earlier theoretical and experimental research, heterogeneous surface reactions can both create ionic concentration gradients and generate local electric fields, leading to interconnected effects. Overall, the charge transport characteristics of the ITO/PFN‐Br/Pt heterostructure, ongoing active dynamics, HER‐related concentration variations, and propulsion that depends on ionic strength all indicate a light‐induced propulsion process. This process involves a significant self‐electrophoretic component and an extra self‐diffusiophoretic contribution.

X‐ray photoelectron spectroscopy (XPS) further reveals surface characteristics before and after the micromotor's motion. The dominant peak at 284.8 eV in the SiO_2_/Ti/ITO/PFN‐Br/Pt micromotor corresponds to the C─C bonds within PFN‐Br, which constitute both the polymer backbone and the flexible side chains that ensure solubility and film‐forming capability (Figure 4a). As presented Figure 4b, the peak at 286.5 eV corresponds to C─N bonds, a characteristic feature of the quaternary ammonium group ((CH_3_)_2_N^+^(C_2_H_5_)─) [59]. The C─C and C─N peaks show almost no change in position or intensity after motion, suggesting that the overall polymer (PFN‐Br) framework is relatively stable under the motion conditions. After motion, a weak additional component appears at 288.2 eV in the C 1s spectrum, which may be assigned to partial oxidation of organic compounds (e.g., C═O). As shown in Figure 4c, the N 1s spectrum after motion is deconvoluted into two peaks. The peak at 402.4 eV corresponds to the quaternary ammonium cation NR_4_^+^, while the new peak at 399.5 eV is attributed to a tertiary‐amine‐like environment (─NR_3_). The content of ─NR_3_ ions increases from 0% to 57.2%, while the NR_4_^+^ decreases to 42.8% after the motion, indicating a substantial change in the local chemical environment of nitrogen. Figure 4d further demonstrates that the characteristic peaks of C─Br (67.5 eV) and Br^−^(68.6 eV) transform into Br─O (74.2 eV) after motion. This change, combined with the oxidized carbon seen in the C 1s spectrum, suggests moderate surface chemical oxidation due to prolonged UV exposure. Importantly, this does not imply substantial degradation of the micromotor structure but rather points to a degree of surface chemical evolution under prolonged UV exposure.

**FIGURE 4:**
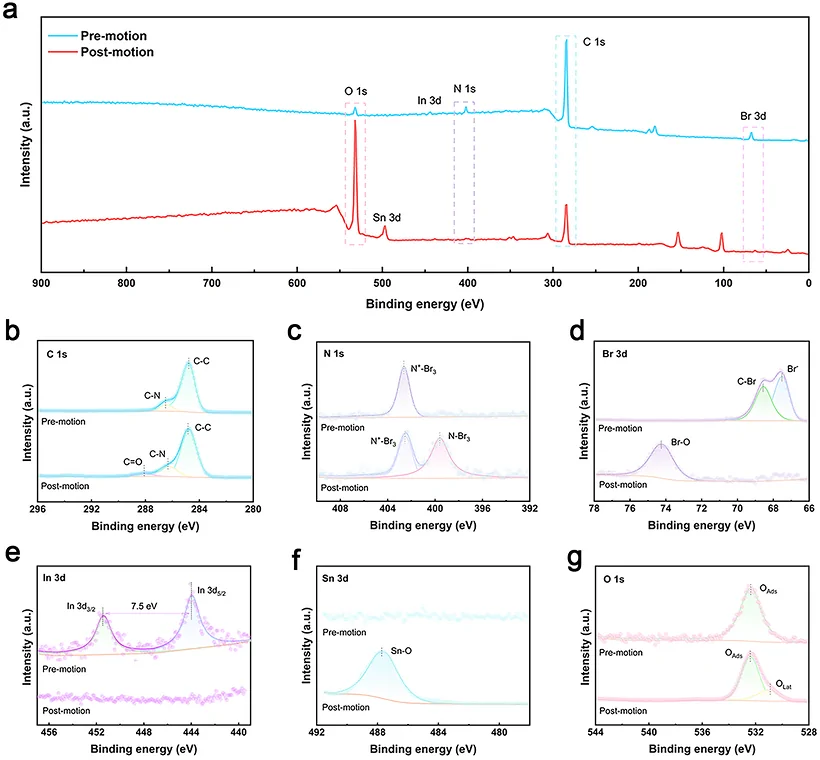
Chemical state and composition of the micromotor surface before and after motion. (a) Full survey scan and high‐resolution spectra of (b) C 1s, (c) N 1s, (d) Br 3d, (e) In 3d, (f) Sn 3d, and (g) O 1s.

Figure 4e shows the In 3d spectrum before and after motion. The characteristic 7.5 eV energy difference between In 3d_3_/_2_ and In 3d_5_/_2_ confirms successful deposition of the ITO layer [60]. After motion, a Sn─O signal at 487.7 eV is detected (Figure 4f), together with a pronounced decrease of the In 3d signal, indicating changes in the local chemical environment and surface composition of the ITO‐containing interface during micromotor motion. However, the post‐motion XPS spectrum by itself does not determine a specific SnO_2_ reduction mechanism or directly reveal the transient carrier‐transfer process. Figure 4g shows that the O 1s spectrum exhibits changes in the relative contributions of surface oxygen species, including lattice oxygen (O_Lat_, 531.0 eV) and adsorbed oxygen (O_Ads_, 532.1 eV), which modulate the surface electric field and reshape the local Stern layer. Here, the O_Lat_ refers specifically to lattice oxygen within the ITO and is consistent with the evolution of the Sn─O‐related chemical environment observed after motion. These modifications suggest that photoinduced motion involves some interfacial chemical changes but does not lead to significant degradation of the micromotor. This is consistent with the previous analysis of the normalized average speed of micromotor (Figure 2e). Furthermore, this phenomenon also stems from the successful fabrication of the heterojunction, as confirmed by the results of the scanning electron microscopy (SEM) analysis shown in Figure 2b. These XPS results show that UV irradiation not only triggers photocatalytic reactions but also causes measurable changes in the interfacial chemistry of the micromotor. These post‐motion changes support the earlier EPR findings, which confirmed the formation of transient reactive oxygen species under UV light. Combined with the photoluminescence (PL) data and the favorable ITO/PFN‐Br energy‐level alignment, these results align with the concept of photoinduced interfacial carrier transfer and related redox reactions occurring within the heterostructured micromotor.

### 2.4 Analysis of Various Configurations’ Motion

The study systematically explored how self‐assembled configurations affect micromotor movement to develop a “shape‐programming” approach for controlling motion. Through modifications in the ultrasonication‐assisted assembly process, structurally identical micromotor units were assembled into dimers (DE) and two typical trimer structures (TR‐I and TR‐II). This allowed for precise control of their collective shapes without altering the fundamental design of each micromotor. Their light‐driven behaviors are shown in Movies S4 and S5. The recorded trajectories over the first 90 s show clear motion patterns depending on configuration (Figures 5a‐c). While individual micromotors moved randomly, DE assemblies mostly followed curved paths (Figure 5a), whereas TR‐I assemblies demonstrated significantly more persistent and nearly straight movement (Figure 5b). In contrast, the TR‐II micromotor moved significantly less (Figure 5c), likely due to its limited symmetry breaking. The configuration‐dependent locomotion results from the interaction among structural asymmetry, propulsion direction, and near‐wall effects. In SL and DE micromotors, the coated “cap” tends to align with the direction of active movement. This is because the hydrodynamic interactions between the solid wall and the microspheres disrupt the symmetric flow field and concentration gradient around the particles, forcing them to orient stably along the wall and preferentially rotate about their axis of motion, resulting in directed motion of the microspheres along the cap direction [61]. In the trimer micromotor, the geometric constraints significantly limit rotational freedom, promoting more consistent directional movement in the TR‐I configuration. This is primarily attributed to the following reasons: Following PFN‐Br coating, the trimer undergoes a slight structural change, differing from the traditional arrangement of three closely packed microspheres. This can be confirmed by high‐resolution SEM (Figure S11a). EDS mapping reveals an uneven distribution of C and N across the microspheres (Figure S11b), aligning with the asymmetric PFN‐Br coverage observed during spin coating. Unlike the more uniform deposition of metallic layers such as Pt, Sn, and In, these variations in PFN‐Br coverage can lead to inconsistent photoactivity in individual microspheres. The interplay of shape‐induced geometric asymmetry, heterogeneous photoactivity, and limited rotational freedom collectively underpins the increased directional motion seen in the TR‐I configuration.

**FIGURE 5:**
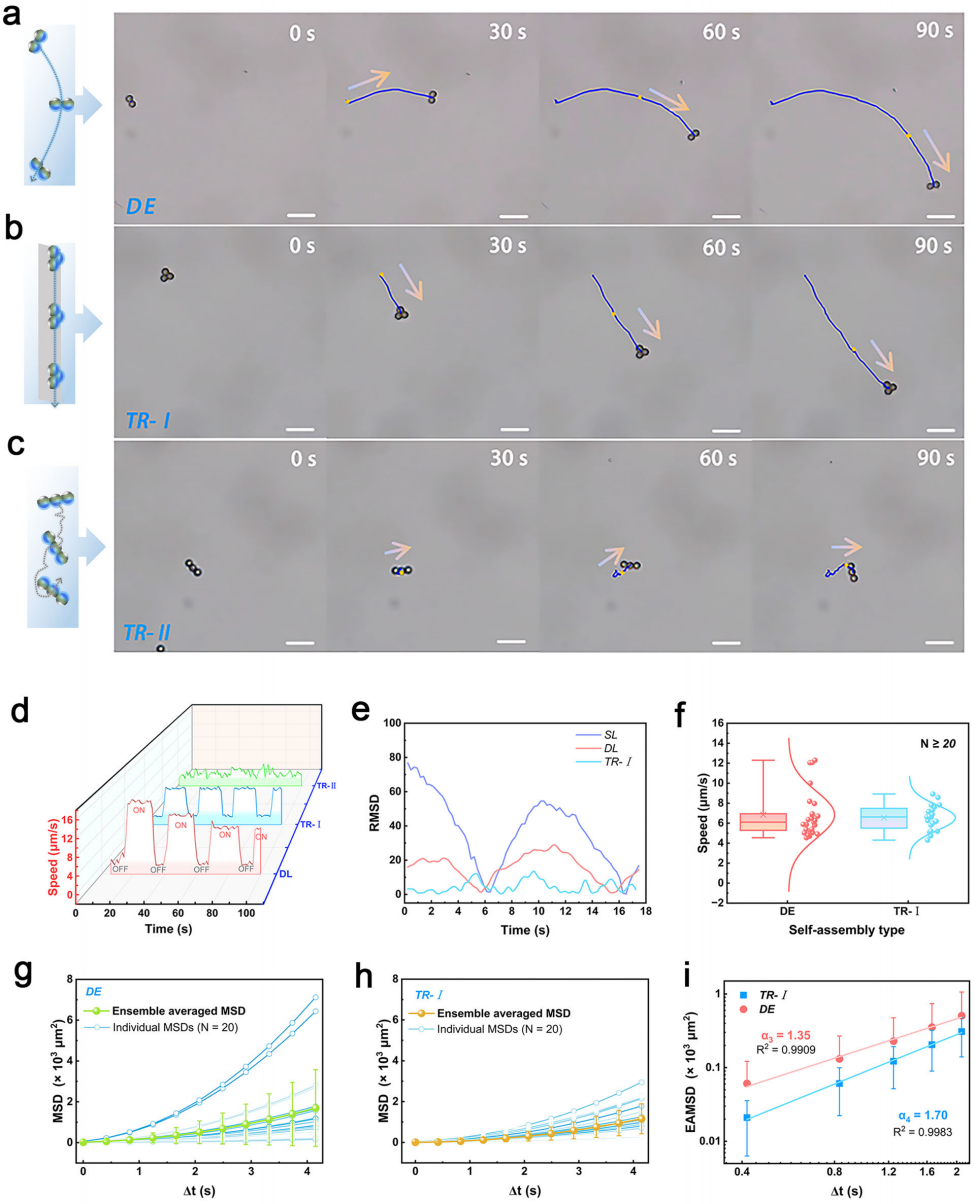
Motion analysis of UV‐driven SiO_2_/Ti/ITO/PFN‐Br/Pt micromotors with different configurations. (a) Dimer (DE) and trimer ((b) TR‐I, (c) TR‐II) micromotors under UV light irradiation at 0, 30, 60, and 90 s, showing their motion trajectories. (Scale bar: 20 µm) (d) Instantaneous speed of the micromotors under UV irradiation on/off (300 mW·cm^−^^2^). (e) Root mean square deviation (RMSD) of the single, dimer, and trimer micromotors. Motion analysis of a single micromotor (with/without ITO layer) under UV illumination at 365 nm. (f) Average speeds of the DE and TR‐I micromotors. Each data point corresponds to an individual micromotor (N = 20 per group), with the average speed computed from a 20 s trajectory. Box plots show the median, interquartile range, and whiskers; individual data points are displayed alongside. (g,h) Individual MSDs and their corresponding EAMSDs of 20 DE and TR‐I micromotors, respectively, as a function of lag time (Δt). Blue lines depict individual MSDs, while green and yellow lines represent the EAMSDs for DE (g) and TR‐I (h) micromotors. Error bars indicate standard deviation. (i) Log‐log plots of EAMSD vs. lag time (Δt) for DE and TR micromotors (N = 20), focusing on the first 2 s of motion. Power‐law fits are shown with solid lines. Error bars denote standard deviation.

Magnetic reorientation experiments further demonstrate that the characteristic motion mode of TR‐I is associated with its assembled configuration rather than simply with a specific cap‐wall orientation. To examine this effect, a 20 nm Ni layer was deposited onto pristine micromotor surfaces to serve as an orientation regulator. The sole effect of an external magnetic field is to induce the TR‐I micromotor to alter its configuration and direction of movement, as documented in Figure S12a and Movie S6. Experimental results demonstrate that the orientation of movement is governed by local concentration gradients determined by initial photoinduced reactions on the microsphere. There are only two linear propulsion modes: either two microspheres propelling a single microsphere, or vice versa. Herein, the magnetic field manipulation merely tunes the direction of movement and does not alter the propulsion mode. Furthermore, trimer flipping experiments triggered by a magnetic field verify persistent linear locomotion irrespective of the relative position of the cap‐wall, which further corroborates the feasibility of shape programming for the regulation of the rectilinear motion of micro‐nanomotors. Moreover, magnetic‐triggered trimer flipping experiments verify persistent linear locomotion irrespective of the “cap” away from or toward the wall, further corroborating the feasibility of shape‐programming for linear motion regulation of micromotors. In addition, the locomotion of “upright” TR‐I micromotors was investigated, as shown in Figure S12b and Movie S7. This configuration is induced by a magnetic field, then exposing it to UV light and immediately removing the field. The driving force here comes only from photoinduced reactions. Results reveal that the “upright” TR‐I retains locomotion under UV irradiation and movement orientation toward the coated side. Nevertheless, its locomotion speed is relatively limited at merely 1.01 µm s^−^^1^ in comparison with the normal TR‐I configuration of 2.82 µm s^−^^1^ (Figure S13). The mechanism of upright TR‐I movement here is consistent with that of SL and DE configurations. In summary, trimers can move regardless of their conformation. Normal TR‐I trimers tend to move in a relatively straight line, whereas upright TR‐I do not necessarily do so.

Additionally, as shown in Figure 5d, during alternating UV light on/off cycles, both the DE and TR‐I configurations of the micromotor exhibited immediate speed switching between active propulsion and Brownian diffusion, consistent with previously reported PEC‐driven micromotor systems [30]. Propulsion performance depends strongly on the assembled configuration. As presented in Figure S14, the DE micromotors exhibited the highest speed of 9.65 µm s^−1^, consistent with previous findings that asymmetric multiparticle assemblies amplify catalytic field gradients and produce stronger net thrust [62]. However, its directional motion control is inferior to that of the TR‐I configuration, which can maintain a speed of 7.33 µm s^−1^. Conversely, the TR‐II configuration exhibited markedly reduced propulsion due to symmetric catalytic surfaces that diminish the effective thrust vector—consistent with reports that excessive symmetry in multi‐particle swimmers suppresses net propulsion. This demonstrates that this approach to controlling the directional motion of micromotors through “shape programming” is highly feasible.

The corresponding root‐mean‐square deviation (RMSD) analysis in Figure 5e shows that the single micromotor displays large positional fluctuations due to low inertia and limited directional constraints. In contrast, the DE micromotor achieves improved directionality [48]. The TR‐I micromotor shows the lowest RMSD, indicating excellent trajectory stability, consistent with prior observations in Movie S5. The MSD analysis further confirms that self‐assembly gradually improves the directional persistence of light‐driven movement from SL to DE and TR‐I, highlighting the effectiveness of shape‐programming regulation in controlling micromotor locomotion (see Figure S15 and Supporting Information for detailed analysis). To assess the repeatability and reliability of configuration‐dependent locomotion, statistical analyses were performed on populations of independently tracked micromotors (Figures 5f‐i). As shown in Figure 5f, the propulsion speeds of DE and TR‐I were statistically analyzed using at least 20 independent micromotors per configuration, with each data point indicating the average speed of an individual micromotor over a 20 s trajectory. The resulting distributions reveal distinct but partially overlapping speed (approximately 5–8 µm s^−^^1^) populations, indicating that the difference in propulsion performance between DE and TR‐I is in repeatability at the population level rather than being inferred from selected individual trajectories. Notably, propulsion speed alone does not fully explain the configuration‐dependent movement, as DE shows relatively high speeds but mostly curved paths, while TR‐I tends to move more in a near‐linear fashion. This difference is further detailed by the MSD analysis of 20 independently tracked micromotors for each type (Figures 5g,h). Both DE and TR‐I display superlinear EAMSD behavior, indicating active propulsion beyond simple diffusion. Fitting the EAMSD with a power law over the first 2 s gives α_3_ = 1.35 for DE and α_4_ = 1.70 for TR‐I (Figure 5i). The higher α value for TR‐I suggests a longer duration of persistent active motion, aligning with its more linear trajectories, while DE's lower exponent corresponds to its curved paths. Overall, the population speed distributions and MSD analysis show that shape programming influences different aspects of micromotor movement: DE tends to be faster but less directionally persistent, whereas TR‐I displays more directed motion. These consistent differences, observed across multiple tracked micromotors, support the idea that shape programming at the configurational level governs the distinct locomotion behaviors. Additionally, these micromotors are capable of transporting cargo efficiently. As shown in Figure S16 and Movie S8, exposure to UV light enables the micromotors to load and transport the cargo, whereas it causes them to stop transporting and unload without light. Concurrently, this structure‐function coupling provides a powerful strategy for engineering optically programmable micromotors that allow precise motion control and directional cargo manipulation at the microscale. Overall, based on the findings above, this shape‐programming‐enabled configurational regulation differs from the recently reported geometry‐programmable light‐driven micromotors. In the latter method, the 0D–3D architectures are deliberately assembled using dimension‐specific fabrication techniques, suggesting that the motion features are mainly determined by the inherent geometry designed into each individual micromotor [63]. In contrast, the “shape‐programming” strategy keeps the same core Janus micromotor as the active unit and controls movement at the assembly level. By adjusting the number, orientation, and spatial layout of the micromotors, it changes their collective symmetry and propulsion vector balance, resulting in different behaviors such as curved, linear, or weak propulsion states. This is achieved without altering the intrinsic geometry of the individual micromotors. Hence, instead of designing geometry during fabrication, this method offers a shape‐programming configurational regulation approach. It provides a complementary way to diversify light‐driven movement through the collective arrangement of active micromotor building blocks.

In this work, “micromotor” specifically refers to a single, light‐driven Janus active unit, while “microrotor” describes a rotational assembly consisting of two interacting particles, at least one of which is an active micromotor. By precisely regulating the assembly behavior of individual micromotors, two distinct types of microrotors (DE‐RI and DE‐RII) were successfully constructed, and their formation pathways were systematically shown in Movie S9 and S10. When dispersed in the TEOA/H_2_O solution, the micromotors exhibited only Brownian motion without light. Under UV irradiation, PFN‐Br absorbed photons and generated excitons, some of which recombined radiatively to produce blue light [38], enabling real‐time tracking. Notably, the photocatalytic reaction occurs on the active hemisphere (the ITO/PFN‐Br/Pt side) with UV light, continuously generating chemical products in the surrounding solution and forming a stable concentration gradient. This gradient field drives adjacent inert SiO_2_ particles (or SiO_2_ side) to migrate toward the active hemisphere via the diffusion‐swimming effect, where they are mutually attracted, thereby providing the key self‐assembly driving force for the formation of microrotors. A similar mechanism has already been verified in TiO_2_/SiO_2_ dimer system [54].

Furthermore, as illustrated in Figures 6a,b, both DE‐RI and DE‐RII microrotors undergo three typical stages during their formation: approaching, attracting, and rotating. DE‐RI microrotors are composed of two actively propelled micromotors, each contributing to propulsion and mutual capture. Their assembly is more challenging than that of DE‐RII microrotors, which form when an active micromotor traps a passive silica particle. To better understand the dynamical mechanism behind microrotor formation, the relationship between the interparticle distance L and the contribution factor ρ of each particle is analyzed (Figures 6c,d). Here,

(3) $$L=\sqrt{{\left(x_{1}-x_{2}\right)}^{2}+{\left(y_{1}-y_{2}\right)}^{2}}\;$$

(4) $$\rho =\frac{v_{i}}{\sqrt{v_{1}^{2}+v_{2}^{2}}}\;\;\left(i=1,2\right)$$

where (*x*_1_,*y*_1_) and (*x*_2_,*y*_2_) denote the two coordinates of micromotors in the plane, *v*_1_ and *v*_2_ are their instantaneous speeds. The ρ is the speed correlation coefficient. As illustrated in Figure 6c, the ρ value of DE‐RI microrotors increases markedly as *L* decreases from 200 to 150 µm, fluctuating around $\frac{1}{\sqrt{2}}\left(\approx 0.7\right)$. This transition indicates that the two micromotors evolve from independent swimmers into a cooperative, dynamically bound pair, formed primarily through mutual capture. Similarly, their speeds exhibit coupled fluctuations due to interparticle interactions. However, for DE‐RII microrotors (Figure 6d), the active micromotor maintains ρ ≈ 1, while the passive silica particle remains close to ρ ≈ 0. This confirms that this type of microrotor mainly forms when the passive particle is adsorbed by the active micromotor—consistent with the higher formation probability observed experimentally.

**FIGURE 6:**
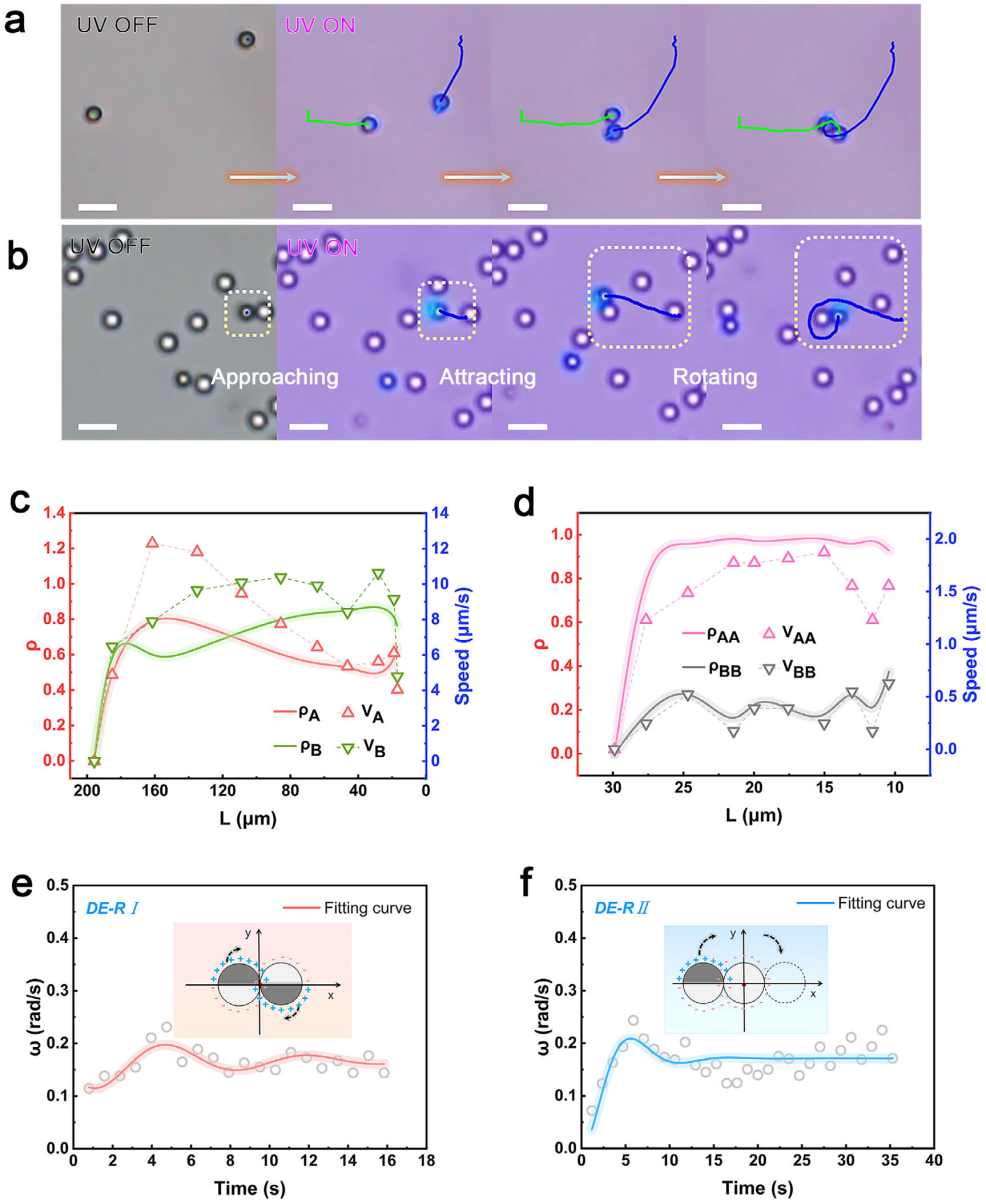
Formation and dynamics of catalytic microrotors. Time‐lapse images show the self‐assembly and rotation of two distinct microrotor types: (a) DE‐RI and (b) DE‐RII. The corresponding analysis of their translational motion during assembly is presented in (c) DE‐RI and (d) DE‐RII, showing the speed (blue axis) and the root mean square speed (red axis) as a function of inter‐motor distance. The rotational dynamics are quantified in (e,f), which plot the angular speed over time for the DE‐RI and DE‐RII rotors, respectively. (In the insert, the gray area represents the coated side, and the white area represents the SiO_2_ side).

Under continuous UV light, DE‐RI microrotors consistently rotate around their common center of mass, whereas DE‐RII microrotors rotate around the passive particle. The persistent rotation arises from asymmetric chemical gradients, misaligned propulsion axes generating active torque, and hydrodynamic coupling. These mechanisms are well‐documented in active colloidal rotors and catalytic systems dimers [64, 65]In both configurations, the angular speed ω increases rapidly within the first few seconds of illumination and stabilizes near ∼0.15 rad s^−^^1^ (Figures 6e,f), as calculated by the following equation:

(5) $$\omega \;\left(t\right)=\omega_{ss}+A\left(1-e^{-t/\tau_{1}}\right)e^{-t/\tau_{2}}$$

where ω_*ss*_ is the final steady‐state angular speed, and *A* denotes the relation to the amplitude of the transient optical drive torque, τ_1_ is the time scale for optical drive torque establishment, and τ_2_ is the time scale for the ion field decay. Such torque–damping balance dynamics are consistent with prior studies on light‐driven microscalerotating devices [66]. Furthermore, the presence of PFN‐Br photoluminescence alongside photocatalytic rotation suggests effective separation of radiative exciton recombination from photoelectrochemical charge use. This simple method allows for the fabrication and utilization of microrotors.

### 2.5 Optical Signal Encoding in Micromotors

The combination of light‐driven movement and PFN‐Br fluorescence allows for externally controlled optical signaling in an actively moving micromotor system. When exposed to UV light, the micromotors demonstrate both photoactivated motion and blue fluorescence. By adjusting the timing of the UV light, the fluorescence can be programmed into specific pulse sequences. Controlling the pulse duration and intervals produces consistent short and long optical signals while the micromotors keep moving. As a potential, these optical pulses were externally programmed into a Morse‐code‐like “SOS” sequence (Movie S11), showing that the fluorescence can serve as a simple, human‐readable signaling tool. This “SOS” pattern is an externally encoded optical message, not autonomous communication between micromotors. From a bio‐inspired standpoint, as shown in Figure S17, this setup highlights the combination of locomotion and tunable optical emission within a single microscale system (see the Supporting Information for details). Overall, the results demonstrate UV‐controlled optical signaling paired with light‐driven movement, not autonomous messaging among micromotors. This feature offers potential for externally programmable optical outputs from moving micromotors and could serve as a foundation for future microsystems that interact with information.

### 2.6 Limitations and Future Perspectives

Despite demonstrating multifunctionality, several obstacles hinder adapting this platform for biomedical use. The SiO_2_/Ti/ITO/PFN‐Br/Pt micromotors move effectively in TEOA/H_2_O (1:9, v/v), but their average speed drops significantly to about 1.64 µm s^−^^1^ in pure water. This emphasizes TEOA's crucial role as a sacrificial hole scavenger in maintaining efficient photoinduced propulsion. However, dependence on such a reagent and the complexity of physiological media limit their direct application in biological settings. Additionally, the system's reliance on UV light for excitation poses further challenges, as limited tissue penetration and potential phototoxicity hinder in vivo use. The current platform should be seen primarily as a foundation for integrating light‐driven movement, shape‐programmable regulation, and controllable optical outputs. It is not yet ready for direct use as a biomedical or autonomous communication microrobotic system. Future efforts might aim to shift the photoresponse into the visible spectrum, enhancing optical access in biological tissues while reducing dependence on UV light. Enhancing charge separation and catalytic efficiency at interfaces could enable propulsion with lower light intensities and potentially eliminate the need for sacrificial reagents. Simultaneously, combining environmentally responsive optical control with local sensing and feedback systems might transition from external signaling to autonomous sensing and response. Achieving consistent operation in physiologically relevant media, alongside thorough assessments of colloidal stability, cytocompatibility, and phototoxicity, will be crucial for advancing this potential platform into adaptive, information‐interactive microrobotic systems suitable for biological conditions.

## 3 Conclusion

In summary, we created a multifunctional SiO_2_/Ti/ITO/PFN‐Br/Pt Janus micromotor platform that supports light‐driven movement, shape‐based configuration control, rotational motion, and fluorescence‐based optical signaling. ITO's inclusion aligns well with PFN‐Br's energy levels, enabling better hole extraction and charge transfer at interfaces, which promotes photoinduced reactions and active propulsion. Increasing ionic strength notably reduces propulsion, indicating a strong self‐electrophoretic effect, alongside a chemical gradient‐driven self‐diffusiophoretic contribution. Importantly, the motion can be controlled at the assembly level by adjusting the number, orientation, and arrangement of the micromotors, without changing their individual geometry. These shape‐programming affect overall symmetry and propulsion vectors, producing varied speeds, trajectories, and directions. Population speed and MSD analyses confirm the consistency and robustness of these varied locomotion behaviors. Self‐assembly also enables rotational microrotor states, broadening the accessible modes of light‐driven motion. Beyond just movement, PFN‐Br's inherent blue fluorescence produces an optical signal during active motion. By modulating external UV excitation over time, programmable fluorescence pulses can be created, including a Morse‐code‐like “SOS” pattern as a potential example. Although these innovations are promising, future practical applications will need to reduce reliance on sacrificial reagents and short‐wavelength photoexcitation, while enhancing propulsion efficiency and robustness in more complex, real‐world environments. Overall, this research highlights shape‐programming‐enabled configurational control as a valuable approach to diversify light‐driven micromotor movement. It also shows how this method can be combined with controllable optical output, laying a versatile foundation for developing multifunctional, information‐interactive micro‐ and nanomotor systems.

## Author Contributions

**Yuyang Zhang** was responsible for the experiments and data analysis and wrote the original manuscript. **Wenyang Huang** participated in data analysis and manuscript revision. **Xinwen Gao** helped prepare the micromotors. **Yuhan He** conducted some of the tests. **Tingting Yu** and **Fei Huang** proposed the experimental idea, designed the experimental protocol, discussed the results, reviewed the manuscript, and obtained financial support.

## Conflicts of Interest

The authors declare no conflicts of interest.

## Supporting information




**Supporting File 1**: advs78303‐sup‐0001‐SuppMat.docx.

**Supporting File 2**: advs78303‐sup‐00010‐MovieS9.mp4.

**Supporting File 3**: advs78303‐sup‐00011‐MovieS10.mp4.

**Supporting File 4**: advs78303‐sup‐00012‐MovieS11.mp4.

**Supporting File 5**: advs78303‐sup‐00013‐SuppMat.pdf.

**Supporting File 6**: advs78303‐sup‐0002‐MovieS1.mp4.

**Supporting File 7**: advs78303‐sup‐0003‐MovieS2.mp4.

**Supporting File 8**: advs78303‐sup‐0004‐MovieS3.mp4.

**Supporting File 9**: advs78303‐sup‐0005‐MovieS4.mp4.

**Supporting File 10**: advs78303‐sup‐0006‐MovieS5.mp4.

**Supporting File 11**: advs78303‐sup‐0007‐MovieS6.mp4.

**Supporting File 12**: advs78303‐sup‐0008‐MovieS7.mp4.

**Supporting File 13**: advs78303‐sup‐0009‐MovieS8.mp4.

## Data Availability

Research data are not shared.
