# Supplementary material for: Catalytic Luminescent Polymer Micromotors With Shape‐Programmed Motion
Source: Adv Sci (Weinh). 2026 Oct 22:e78303. Online ahead of print. doi: 10.1002/advs.78303 (PMC13690988; doi:10.1002/advs.78303)
Supplement: Supplementary file 1 [file ADVS-9999-e78303-s003.pdf]

Supporting Information for

Catalytic Luminescent Polymer Micromotors with Shape-Programmed Motion

Yuyang Zhang, Wenyang Huang, Xinwen Gao, Yuhan He, Fei Huang*, Tingting Yu*

*Corresponding author: Tingting Yu Email: tingtingyu@sjtu.edu.cn

Fei Huang Email: msfhuang@scut.edu.cn

This file includes:

Methods

Figure S1 to S17

Tables S1

Diagram for movies S1 to S11

Other Supplementary Materials for this manuscript include the following:

Movies S1 to S11

Methods

Materials and equipment. High-purity metal and oxide sources, including titanium (Ti, 99.999 wt%), platinum (Pt, 99.99 wt%), and indium tin oxide (ITO, In:Sn = 9:1, 99.99 wt%), were purchased from Zhongnuo New Materials (Shanghai) Technology Co., Ltd. Methanol (99.5%), anhydrous ethanol (99.5%), acetone (99.5%), triethanolamine (TEOA, 98%), and a monodisperse silica microsphere solution (5.0 μm diameter, 2.5% stock concentration) were obtained from Macklin Biochemical Co., Ltd. The organic semiconductors polymer Poly[(9,9-bis(3'-((N,N-dimethyl)-N-ethylammonium)-propyl)-2,7-fluorene)-alt-2,7-(9,9-dioctylfluorene)] dibromide (PFN-Br) was synthesized from its neutral precursor polymer PFN, which was synthesized using standard Suzuki polymerization[1]. Deionized water was used throughout all experiments. Before use, glass slides were ultrasonically cleaned in acetone and ethanol for 10 and 20 min, respectively, followed by three cycles of ultrasonic rinsing in deionized water (10 min each), ensuring a reliably clean substrate surface. Optical imaging was conducted using an inverted microscope (Olympus IX73) with a LUCPLFLN40XPH objective and a 20.7-megapixel color-cooled color camera (DP74-CU). Image acquisition and preliminary processing were performed using the cellSens Entry platform. Particle trajectory analysis was performed in ImageJ, and statistical analysis was conducted in Origin. The micro- and nanostructure morphology and elemental composition were characterized using SEM and EDS (TESCAN GAIA3). X-ray diffraction (XRD) patterns were recorded with an X'Pert Powder diffractometer equipped with an automated multi-sample stage. UV-Vis-NIR spectra were obtained using an Agilent Cary 5000 spectrophotometer, while photoluminescence spectra were collected using an Edinburgh FLS1000 steady-state/transient spectrometer. Fluorescence spectra were collected with an Edinburgh Instruments FS5 spectrofuorometer. Electron paramagnetic resonance (EPR) spectroscopy

was performed using a Bruker EMXplus-6/1 spectrometer to detect hydroxyl radicals (·OH) and superoxide radicals ($\cdot O_2^-$). 5,5-Dimethyl-1-pyrroline N-oxide (DMPO) was employed as the spin-trapping agent. Zeta potential measurements were performed using a HORIBA SZ-100Z DLS analyzer. X-ray photoelectron spectroscopy (XPS) was performed on a Kratos Axis Supra$^+$ system equipped with a monochromatic Al Kα X-ray source, using a spot size of 650 μm and a chamber pressure of 5.0×10^{-10} mbar.

Preparation of micromotor locomotion. Monodisperse SiO_2 microspheres with an average diameter of approximately 5 μm were first dispersed in anhydrous ethanol ($V_{SiO_2\ solution}$: V_{EtOH} = 2:3). A controlled evaporation deposition method (the vacuum chamber was pumped to 3.0×10^{-6} Pa) was used to assemble the microspheres into a densely packed monolayer on glass substrates (Fig. 2a). After complete evaporation of ethanol, a 5 nm Ti adhesion layer was deposited onto the microsphere surface via electron-beam evaporation. Subsequently, a 60 nm ITO layer was deposited onto the SiO_2/Ti microspheres by electron-beam evaporation and annealed at 400 °C for 20 min in a muffle furnace. During annealing, the ITO layer gradually changed from metallic to transparent, indicating successful deposition and oxidation. The polymer semiconductor PFN-Br was dissolved in methanol at a concentration of 2 mg mL^{-1} and spin-coated onto the SiO_2/Ti/ITO microsphere substrate. The spin-coating procedure consisted of a spreading step at 800 rpm for 5 s, followed by film formation at 4000 rpm for 40 s. The coated substrates were then annealed at 120 °C for 20 min to ensure complete methanol removal and enhance the stability of the bulk-heterojunction polymer layer. Finally, a 40 nm Pt layer was deposited onto the PFN-Br–coated microspheres via electron-beam evaporation, yielding fully assembled SiO_2/Ti/ITO/PFN-Br/Pt micromotors. The thickness of the layers (Ti, ITO, Pt layer) here is measured using the quartz crystal microbalance system (QCM) integrated into the electron beam equipment. The fabricated micromotors were released from the glass

substrate by mild ultrasonication and then dispersed in an aqueous TEOA solution (TEOA:H_2O = 1:9) for locomotion testing. Ultraviolet illumination at 365 nm served as the excitation source during motion experiments.

Micromotors motion observation and data analysis. An TEOA/H_2O solution containing micromotors was introduced into the chamber on the microscope slide, which was enclosed by a silicone ring measuring 100 × 100 × 2 mm. Recording motion videos using an optical microscope (Olympus IX73), an ultraviolet laser emitter (THORLABS-DC2200), and a digital camera (Olympus). All videos were recorded using 20× or 40× objectives, with the initial frame rate of 25 frames per second and resolution of 1920 × 1080. To analyze the movement speed using ImageJ, the video is first decomposed into individual frames. The speed is then calculated by tracking the trajectories of the micromotors.

Shape programming assembly experiment. The micromotors were detached from the substrate in the form of single (SL), dimers (DE), and trimers (TR) configuration primarily by adjusting the ultrasonic device's parameters (frequency and duration), rather than by the conventional method of controlling individual micromotors to assemble into multimers. The experiments revealed that at 100% power and an ultrasonic duration of over 2 minutes, the micro- and nano-robots remained almost exclusively single micromotors.

Speed normalization method. This study investigated the motion stability of a single micromotor under ultraviolet irradiation and the effects of photodegradation by performing full-process dynamic tracking of a selected micromotor. The average speed was calculated for each 10s interval, and the average speeed during the first 10 seconds was selected as the reference value for normalization. The calculation formula is: $\eta_t = s_t/s_{ref}$. where s_t represents the average speed over different 10 s intervals, and s_{ref} is the first 10 seconds reference speed. This method eliminates interference caused by differences in the initial

motion performance of individual micromotors and intuitively illustrates changes in the motion performance of a single micromotor under prolonged light exposure.

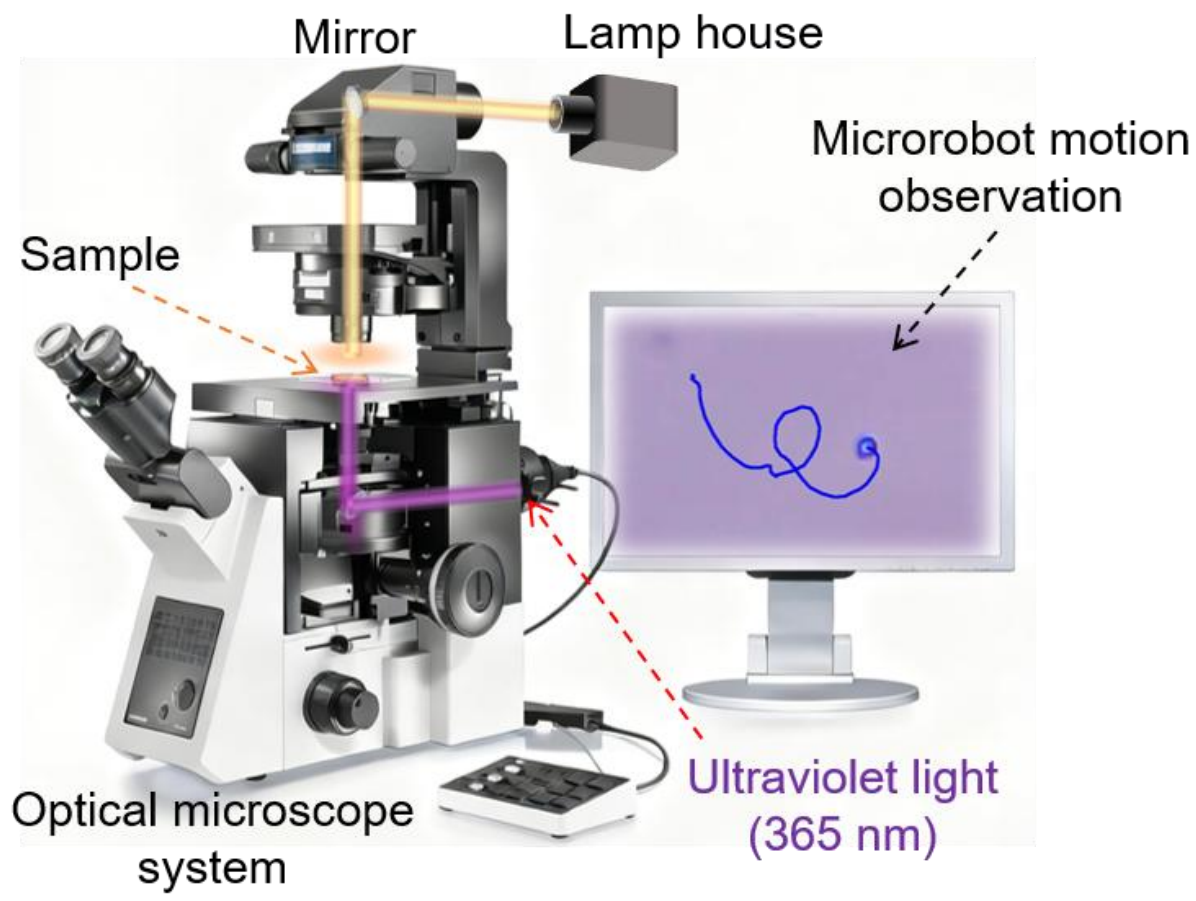

Figure S1. Schematic diagram of the observation setup for the micromotor motion experiment.

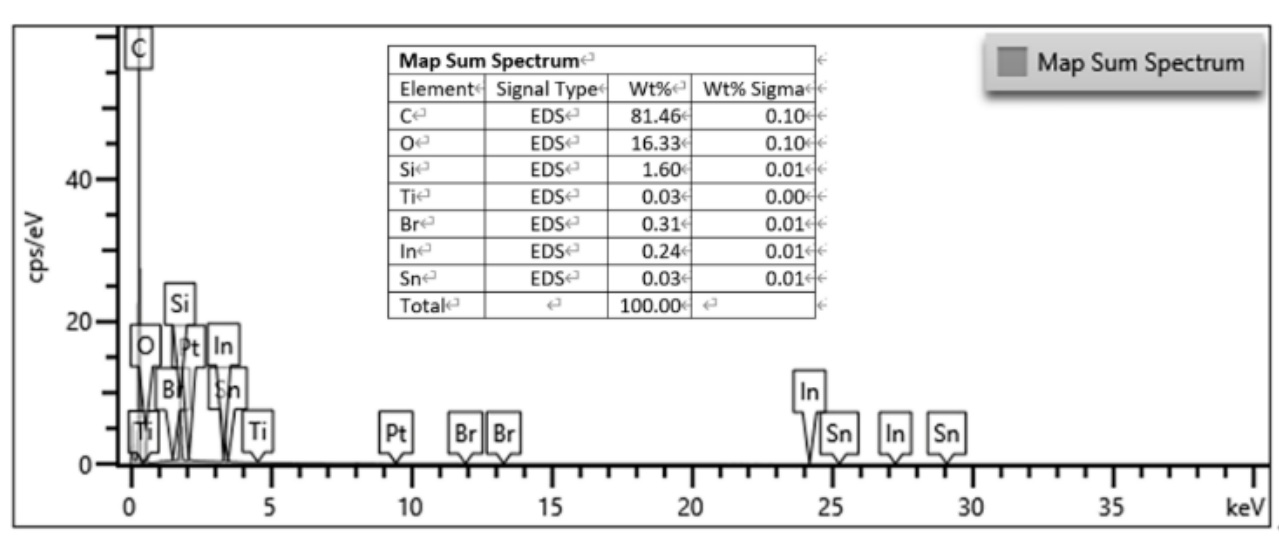

Map Sum Spectrum			
Element	Signal Type	Wt%	Wt% Sigma
C	EDS	81.46	0.10
O	EDS	16.33	0.10
Si	EDS	1.60	0.01
Ti	EDS	0.03	0.00
Br	EDS	0.31	0.01
In	EDS	0.24	0.01
Sn	EDS	0.03	0.01
Total		100.00	

Figure S2. Energy-dispersive X-ray spectroscopy (EDS) spectrum of the SiO_2/Ti/ITO/PFN-Br/Pt micromotor.

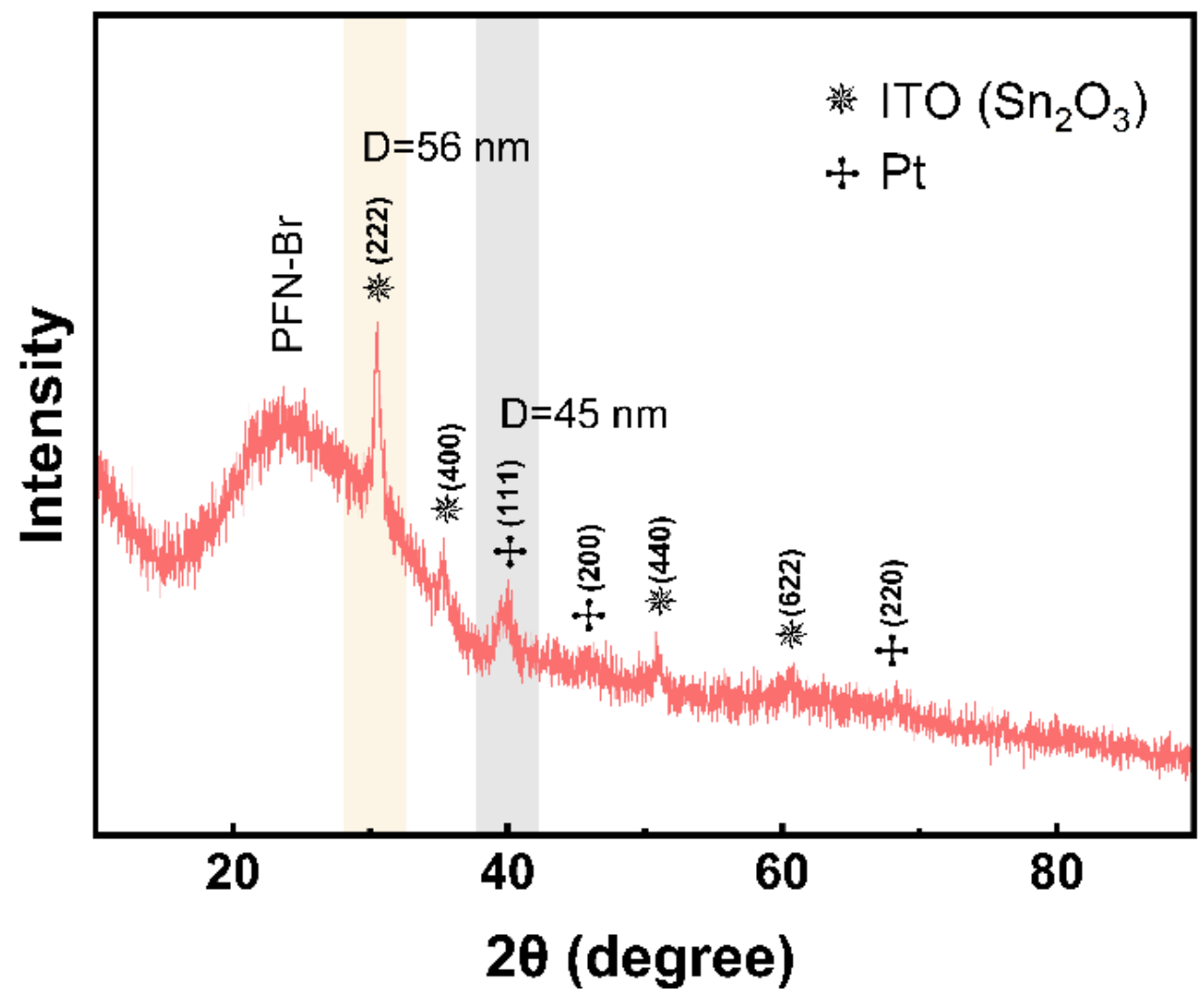

Figure S3. XRD pattern of the SiO_2/Ti/ITO/PFN-Br/Pt micromotor.

The surface phase composition was analyzed using X-ray diffraction (XRD), and the corresponding diffraction pattern is shown in Figure S2. The broad amorphous features are consistent with the incorporation of the PFN-Br polymer layer.[2] Distinct diffraction peaks at 30.5°, 35.5°, 50.9°, and 60.7° correspond to the (222), (400), (440), and (622) planes of crystalline ITO (PDF#06-0416),[3] while peaks at 40.2°, 46.2°, and 68.1° match the (111), (200), and (220) planes of Pt (PDF#04-0802).[4] These results confirm the successful construction of the layered Janus architecture of the micromotors. Scherrer analysis (Eq. S1) estimates average crystallite sizes of ~ 56 nm for ITO and ~ 45 nm for Pt.

$$D = \frac{K\lambda}{\beta \cos\theta} \text{ [5]} \tag{S1}$$

Where D: grain size; K: cherrer constant, a dimensionless constant related to grain shape, crystal plane indices (hkl) and β definitions; λ: the wavelength of the incident X-rays; β: half-width at half maximum of the diffraction peak; θ: prague corner. These nanoscale crystalline domains are expected to improve catalytic activity and contribute to

the efficient propulsion performance of the micromotors.

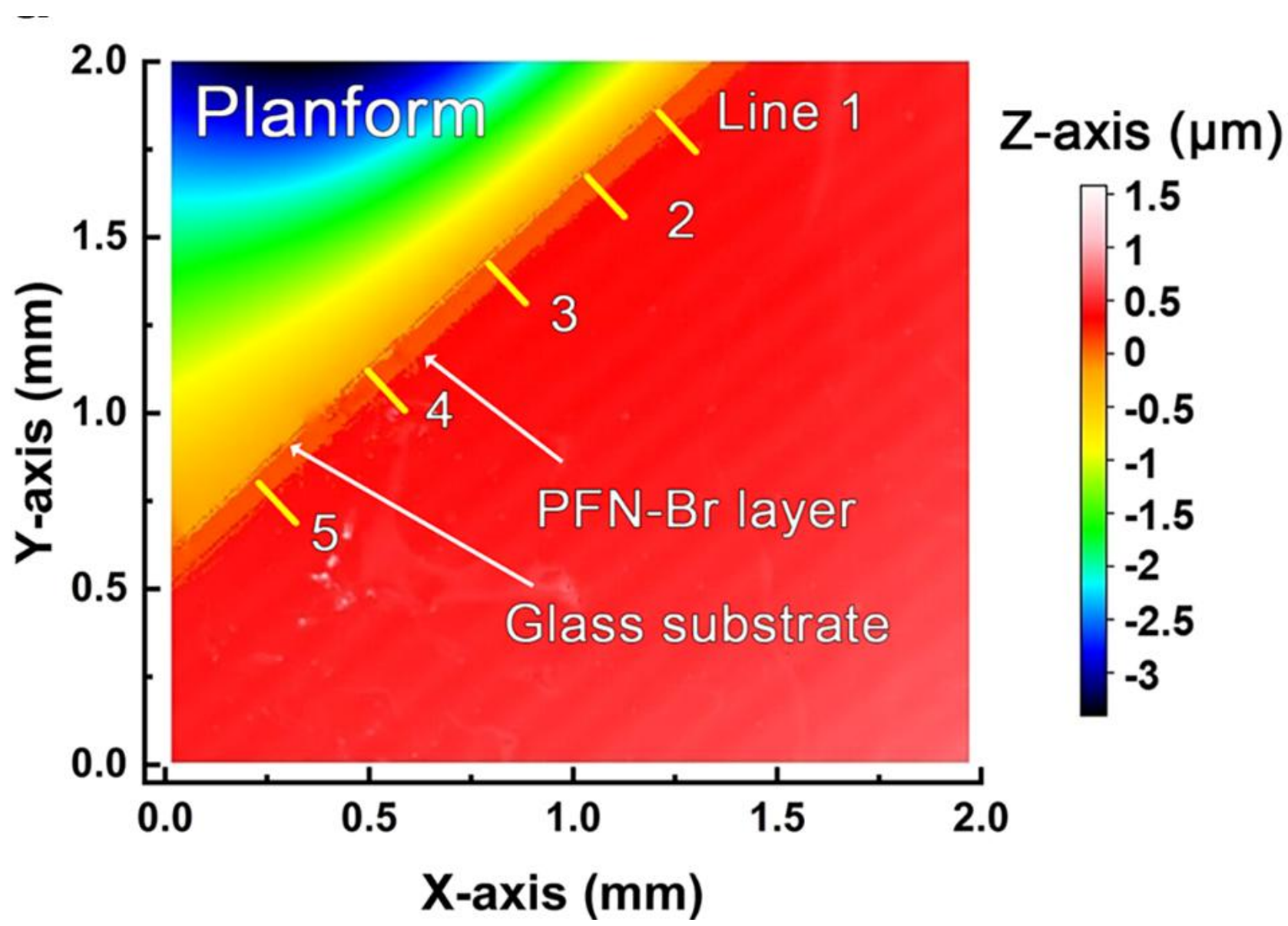

Figure S4. 2D colored contour map of PFN-Br spin-coated layer with five marked cross-section lines.

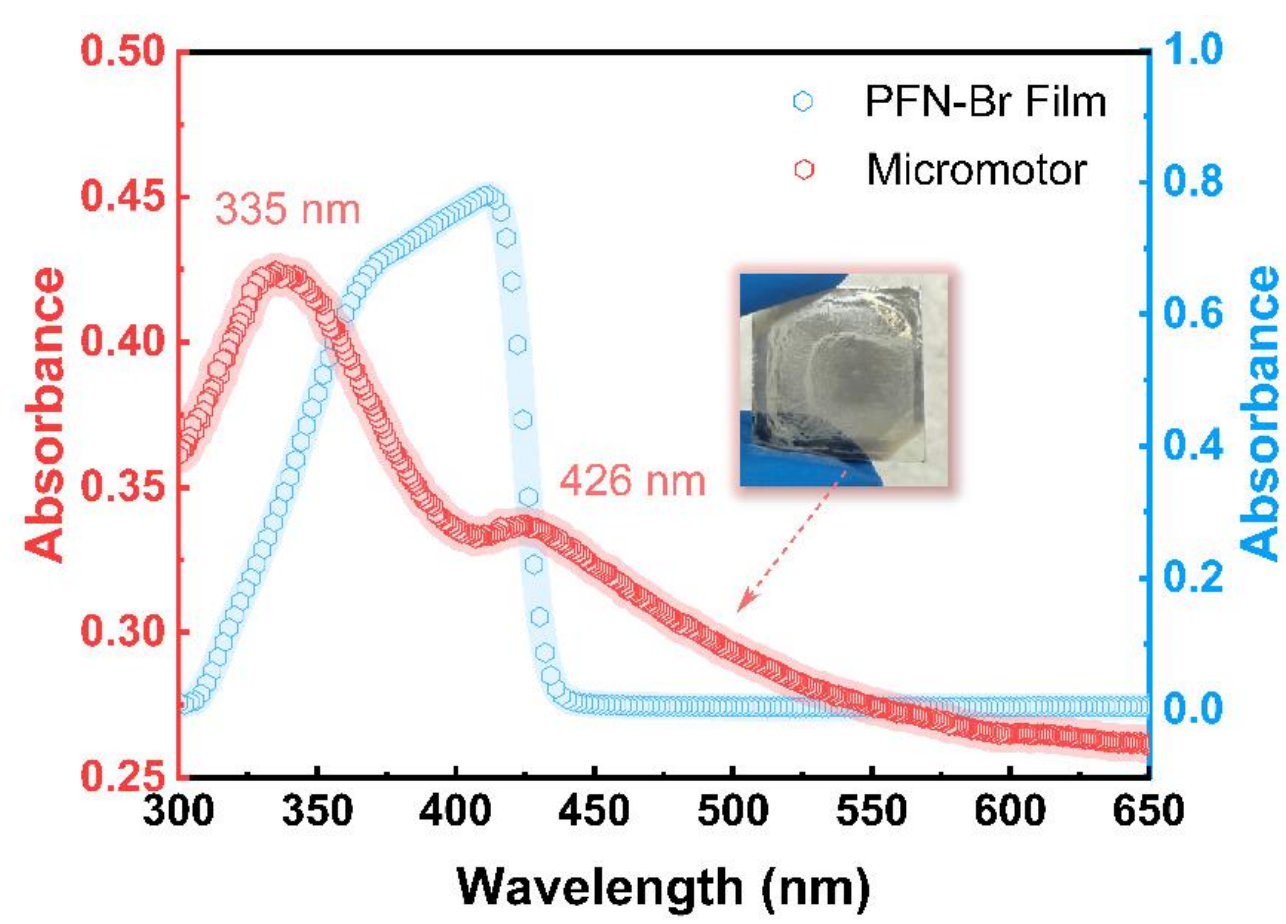

Figure S5. Ultraviolet-visible absorption spectrum of the SiO_2/Ti/ITO/PFN-Br/Pt micromotor.

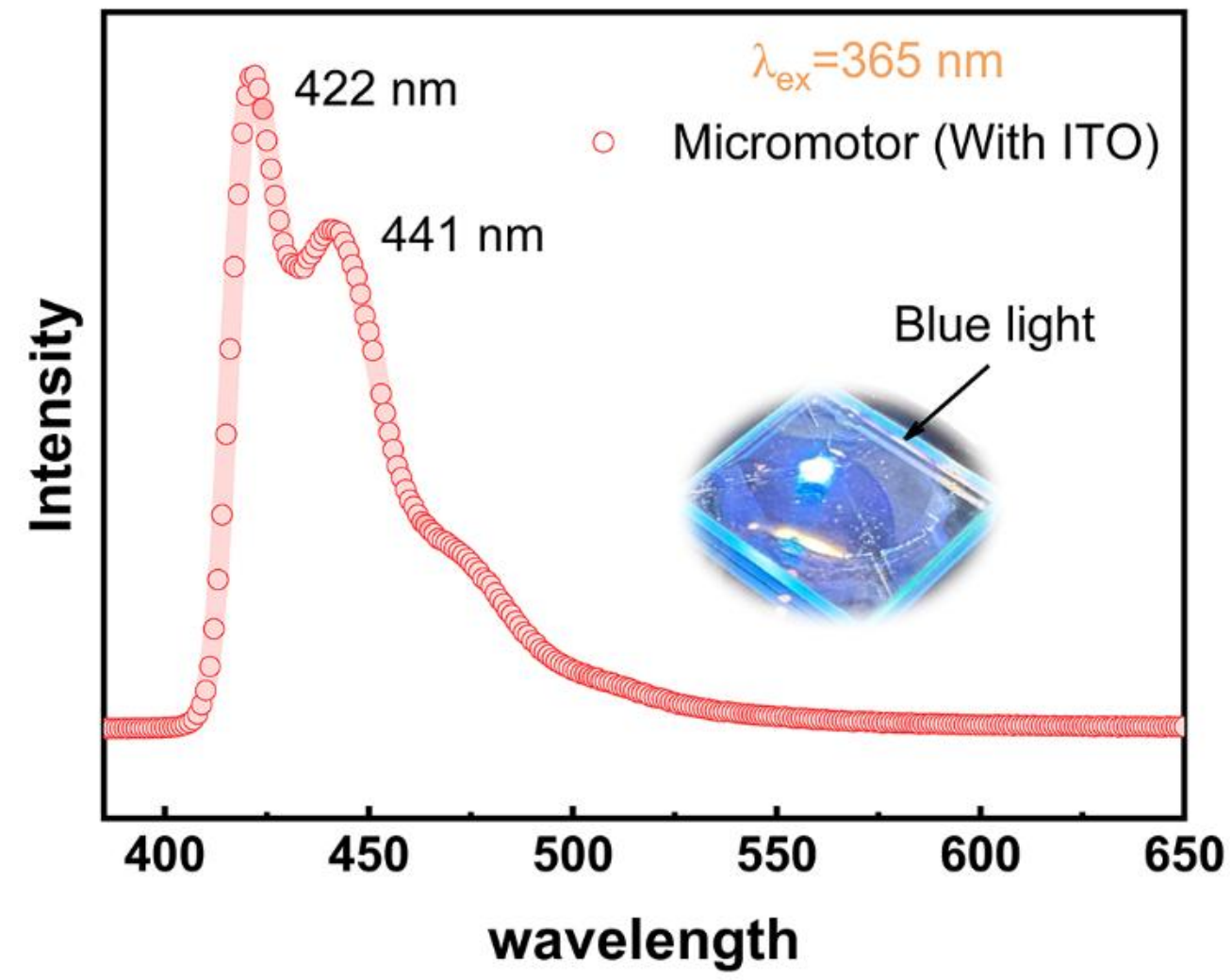

Figure S6. Fluorescence emission spectrum of the SiO_2/Ti/ITO/PFN-Br/Pt micromotor. The excitation wavelength λ_{ex} is 365 nm.

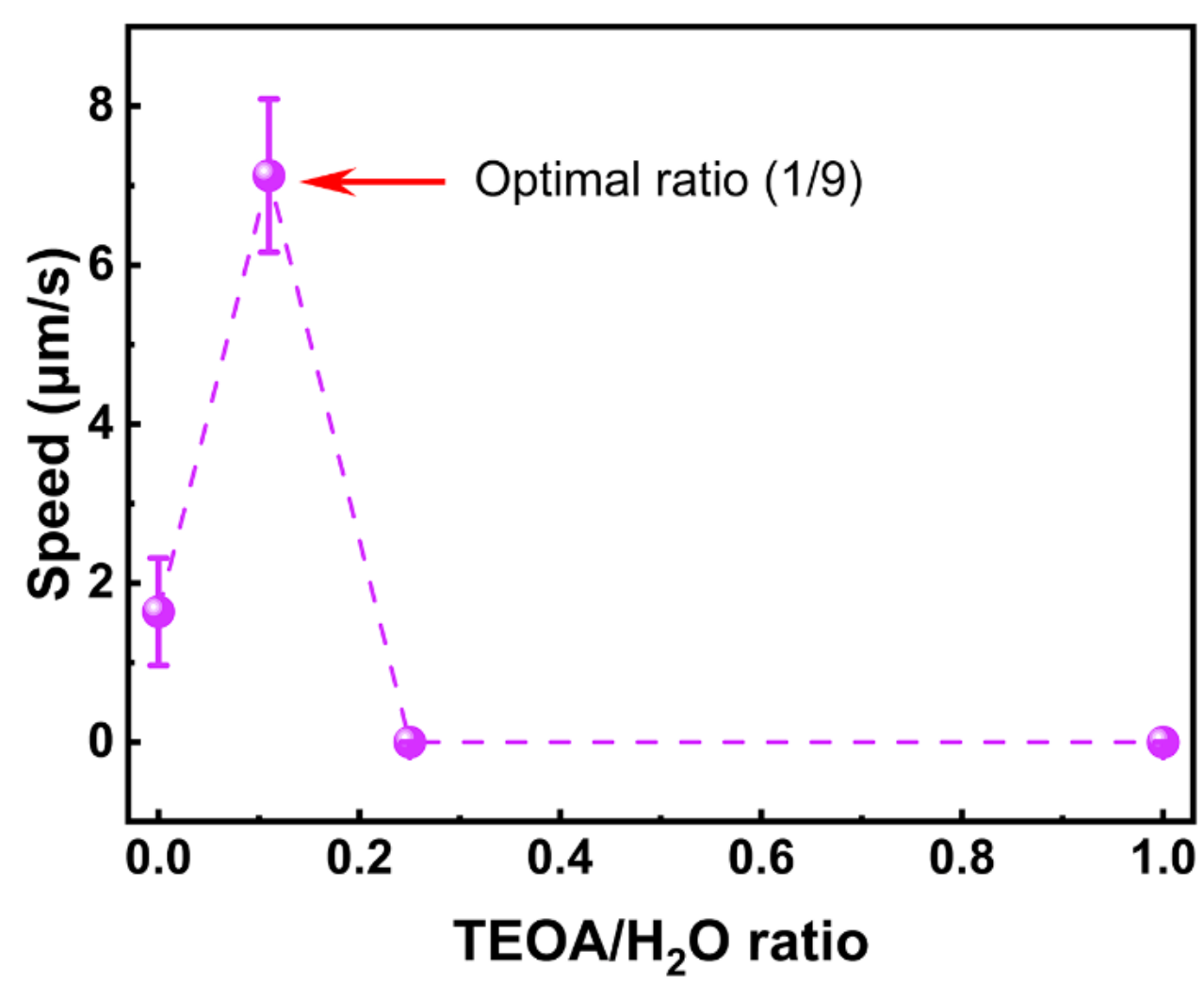

Figure S7. Average motion speed of micromotors at different TEOA/H_2O ratios. Error bars represent the standard deviation.

The propulsion speed depended on the TEOA concentration, peaking at about 7.2 μm s^{-1} with a TEOA/H_2O volume ratio of 1/9. Increasing TEOA further significantly reduced

the movement, with almost no net displacement seen at ratios of 2/8 and 1/1. This suppression at high TEOA concentrations may be associated with changes in the increased solution viscosity caused by the elevated TEOA concentration, which leads to enhanced adhesion between the micromotors and the substrate. Therefore, a TEOA/H_2O ratio of 1/9 was determined to be the optimal condition for micromotor propulsion. Unless stated otherwise, all future experiments involving micromotor movement will use an aqueous solution of TEOA at this concentration.

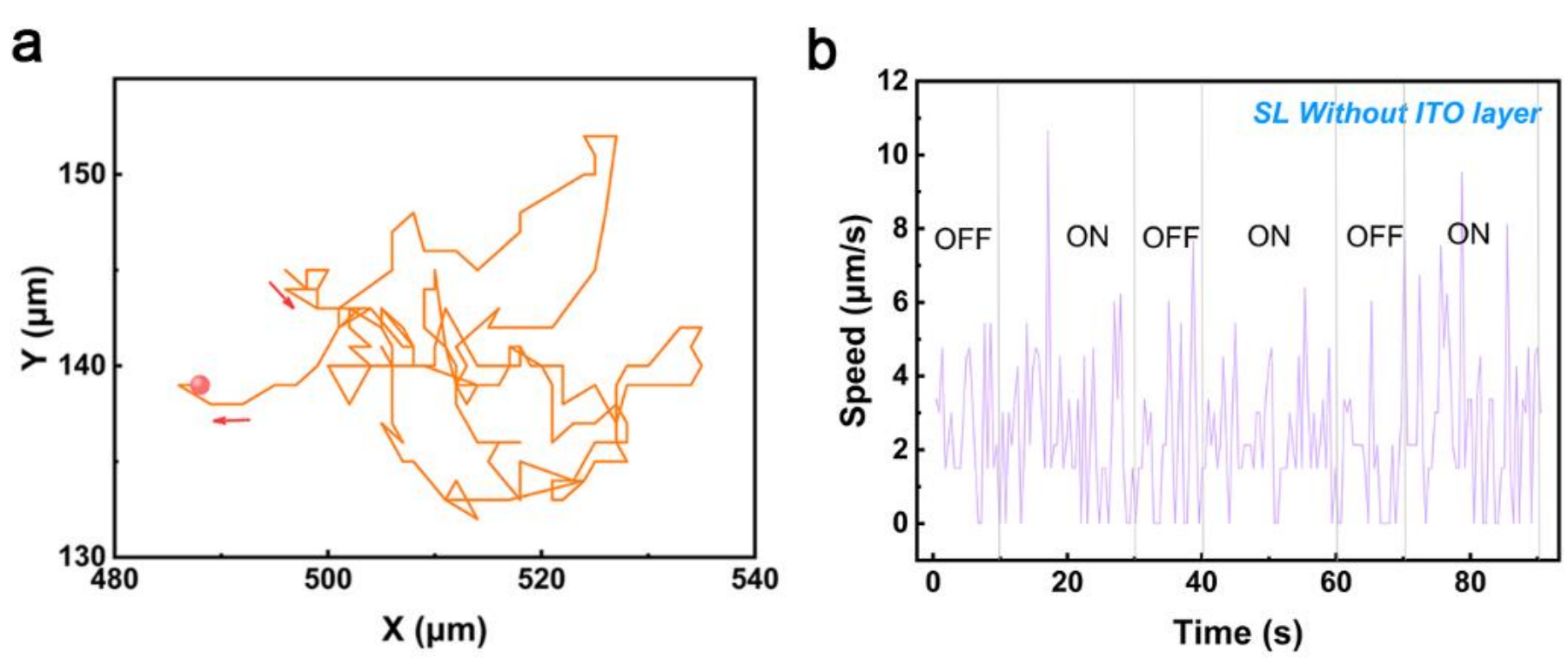

Figure S8. (a) Trajectory of the SiO_2/Ti/PFN-Br/Pt micromotor (without ITO layer) under 365 nm illumination over 20 s. (b) Speed response of the micromotor to cyclic on/off switching of UV illumination.

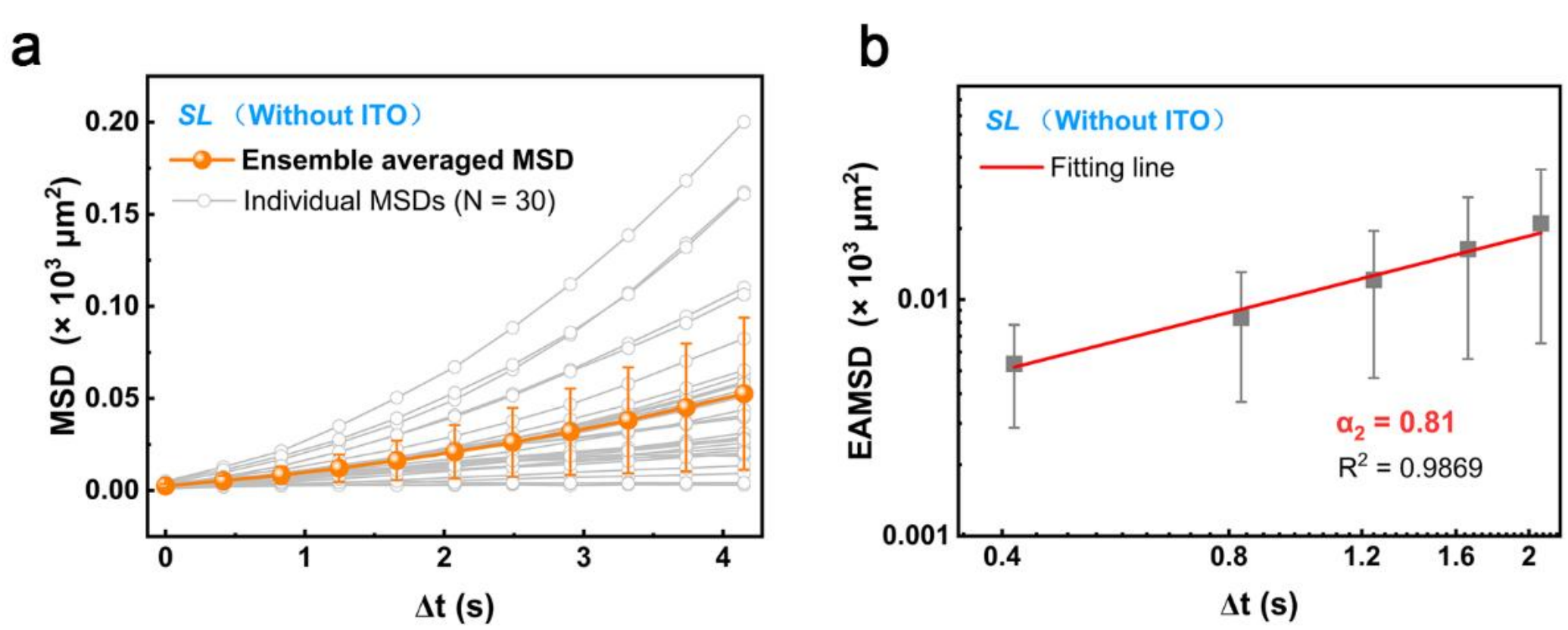

Figure S9. (a) The MSDs of 30 individual micromotors and the corresponding EAMSD are shown as functions of lag time (Δt). Gray lines indicate each individual MSD, while the orange line shows the average EAMSD across all 30 trajectories. Error bars depict the standard deviation. (b) Log-log plot of EAMSD versus lag time (Δt), with the red line indicating a power-law fit over the first 2 seconds. Error bars show standard deviation.

All MSD curves exhibit a consistent upward trend with a frame interval (Δt) of 1.0 s, signifying ongoing displacement over time. The slope of each curve varies significantly with ultraviolet light intensity, emphasizing the importance of light exposure in regulating locomotor activity. At low power levels, Brownian motion prevails due to lack of sufficient activation energy. As the intensity rises from 35 to 75 mW cm^{-2}, the MSD curves steepen, indicating increased movement driven by enhanced photocatalytic reactions. At higher intensities of 150 and 260 mW cm^{-2}, the curves become notably steeper, pointing to a shift from diffusive to directed motion. This suggests that under strong illumination, micromotors maintain better directional stability and exhibit active propulsion. The dependence of MSD on light intensity relates to photochemical kinetic mechanisms: as light intensity increases, the photocatalytic reaction rate accelerates, strengthening the local chemical gradient and driving force, which in turn boosts micromotor speed.

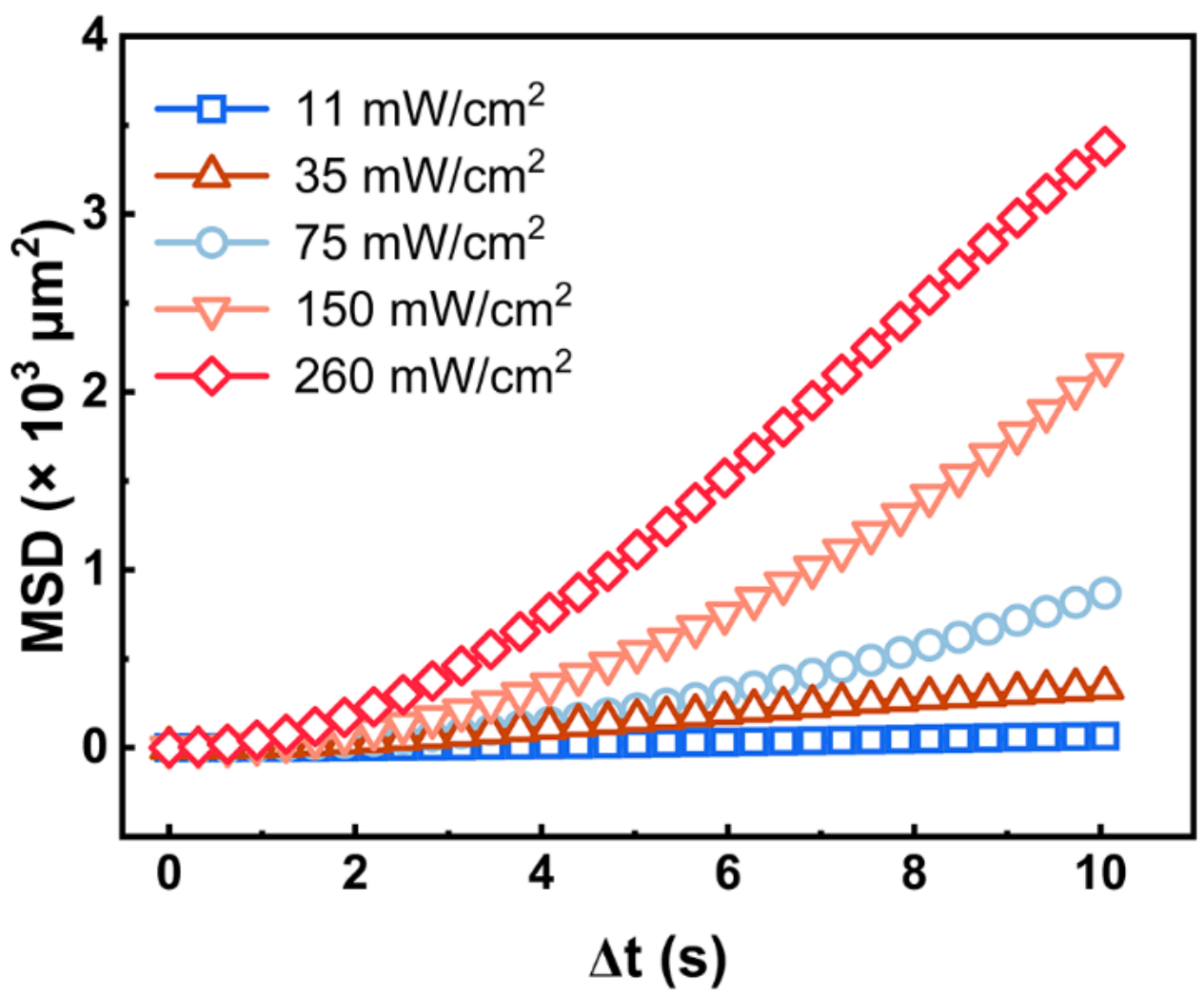

Figure S10. Mean squared displacement (MSD) of the micromotor under UV irradiation on/off conditions.

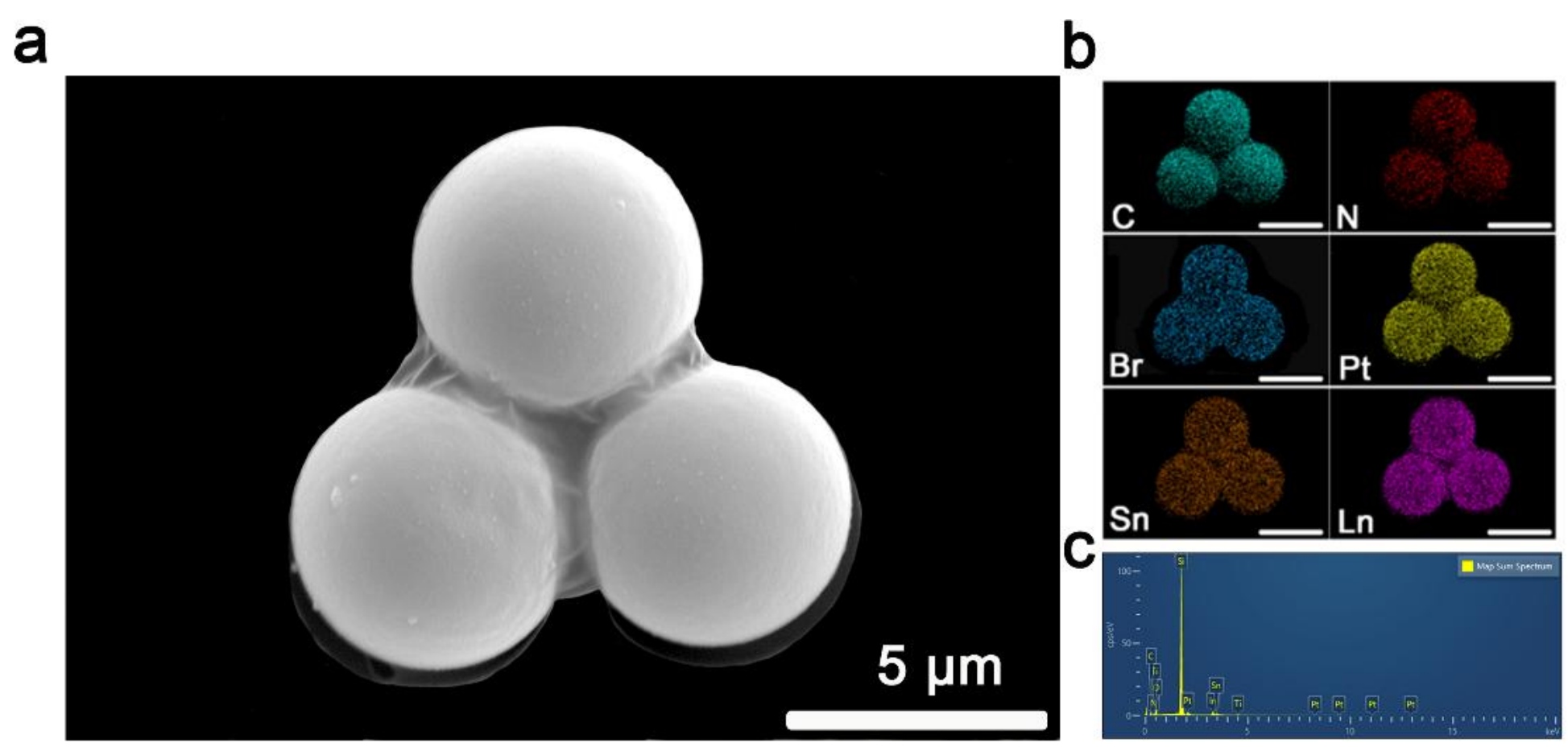

Figure S11. (a) SEM image of a SiO_2/Ti/ITO/PFN-Br/Pt trimer micromotor on a substrate. (b) Corresponding EDS elemental mappings for C, N, Br, Pt, Sn, and In. (c) The EDS spectrum of the trimer micromotor.

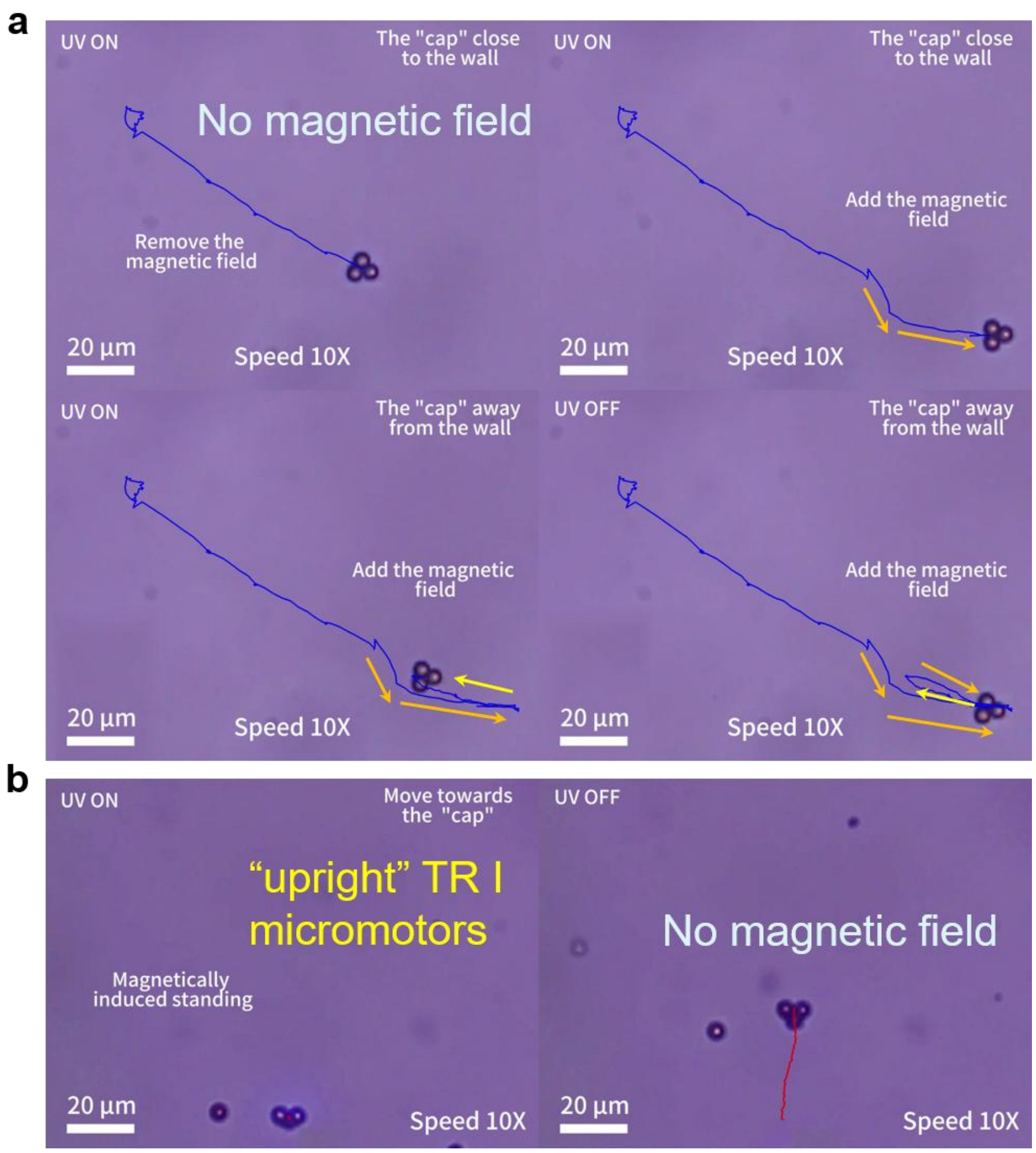

Figure S12. Controllable motion of the SiO_2/Ti/ITO/PFN-Br/Pt/Ni trimer micromotor (Driven solely by a light field). (a) Controllable linear motion trajectory of a normal TR-I trimer micromotor. (b) Motion trajectory of “upright” TR I micromotors

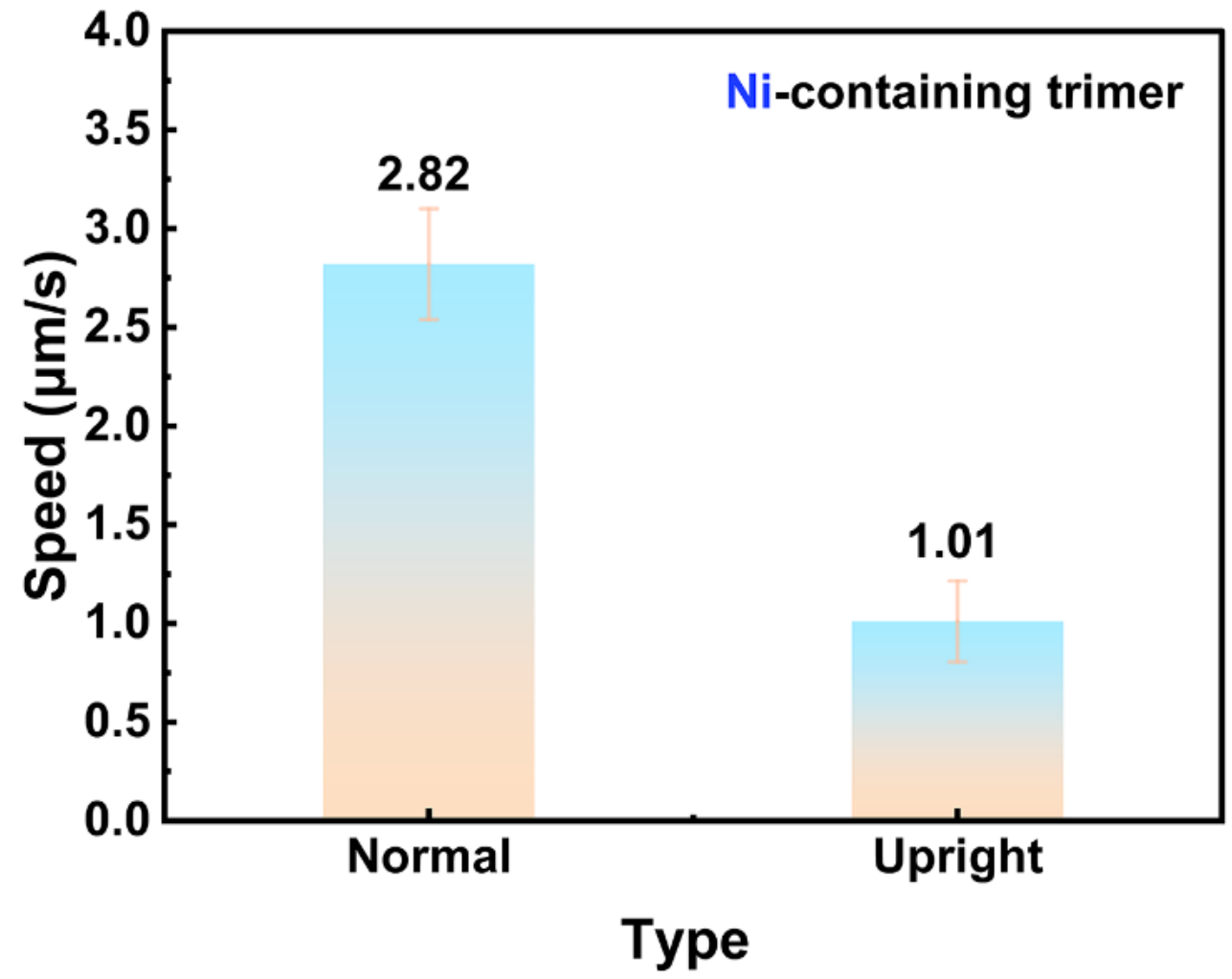

Figure S13. Speed comparison of a SiO_2/Ti/ITO/PFN-Br/Pt/Ni trimer micromotor moving in two different modes. Error bars represent standard deviation.

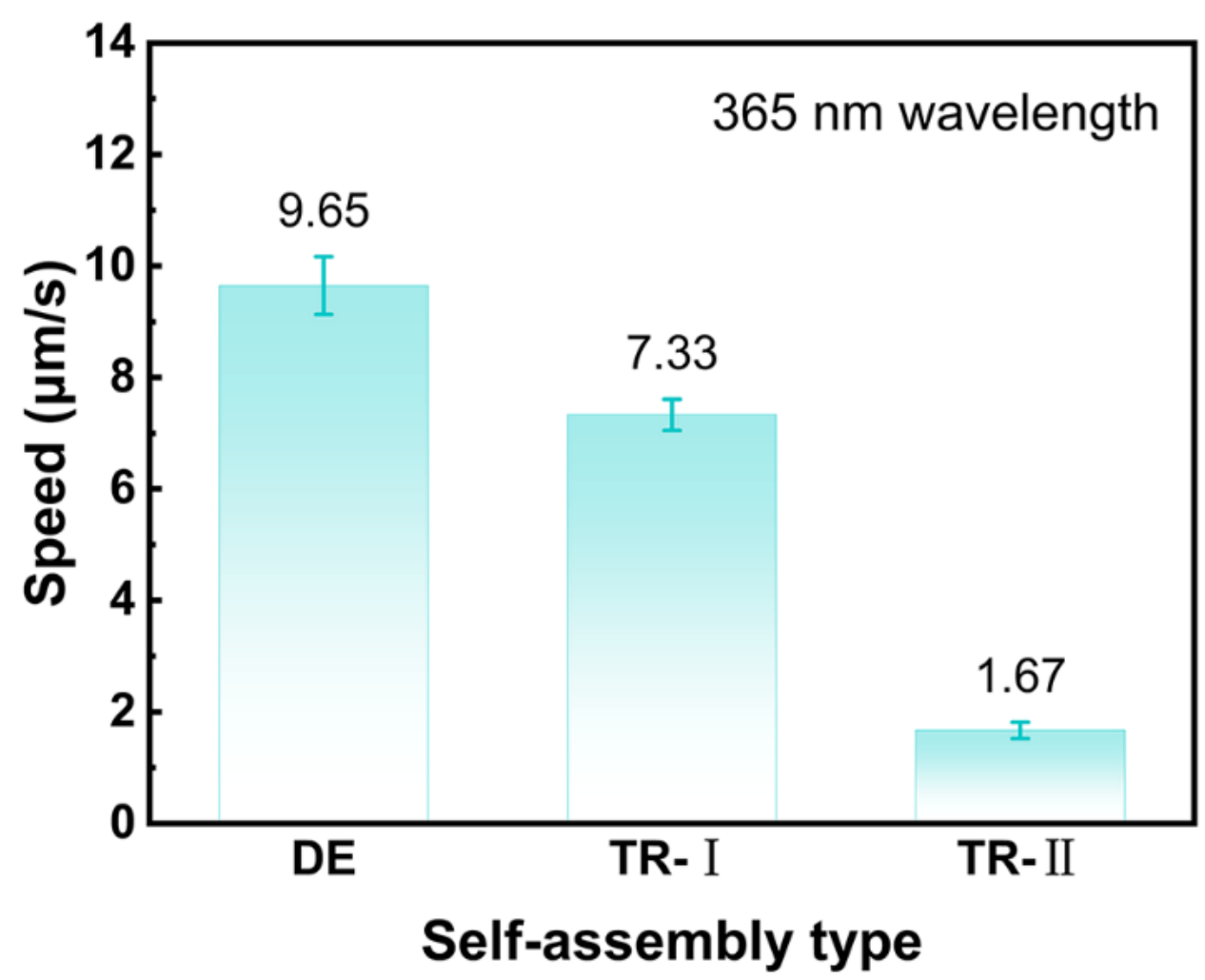

Figure S14. Average velocity histogram of micromotors with different configurations. Error bars represent standard deviation.

The MSD curves with a 0.5 s frame interval in Figure 16a-c quantitatively support these observations. Under UV illumination at 300 mW cm^{-2}, all configurations exhibit a transition from linear to quadratic MSD growth (MSD $\propto \Delta t^2$), confirming active propulsion. The MSD values follow the order SL < DE < TR-I, correlating with increasing

directionality and persistence length. In the absence of UV illumination, all MSD curves revert to nearly linear Brownian scaling with negligible displacement, further demonstrating the necessity of PEC activation for motion. Overall, shape programming can evolve micromotors from weakly directed single units into highly stable trimer assemblies, enabling persistent motility.

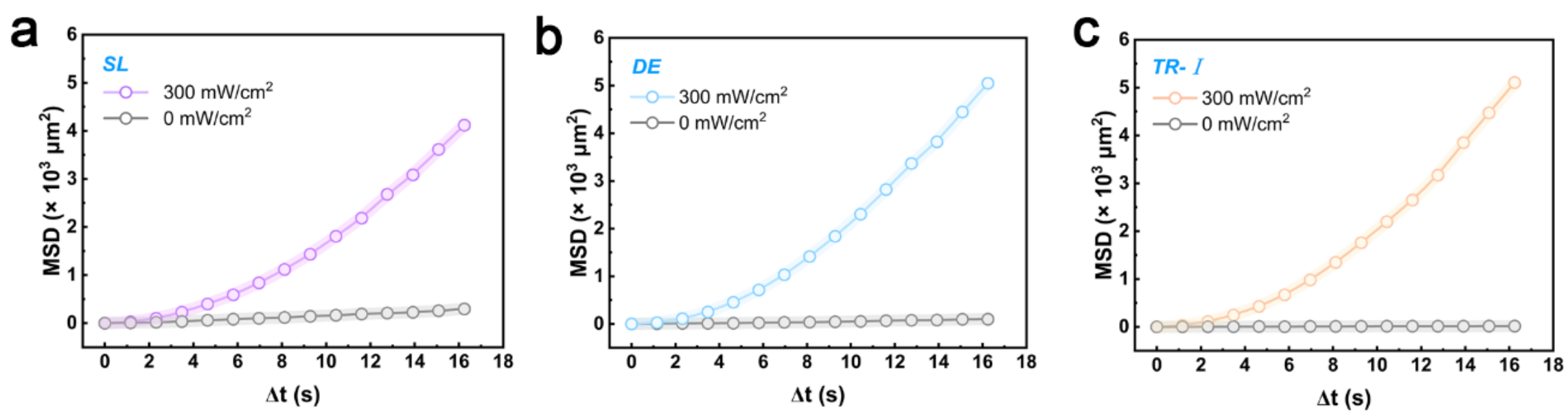

Figure S15. (a-c) Mean square displacement (MSD) of corresponding single, dimer, and trimer (TR-I) micromotors under UV irradiation on/off conditions.

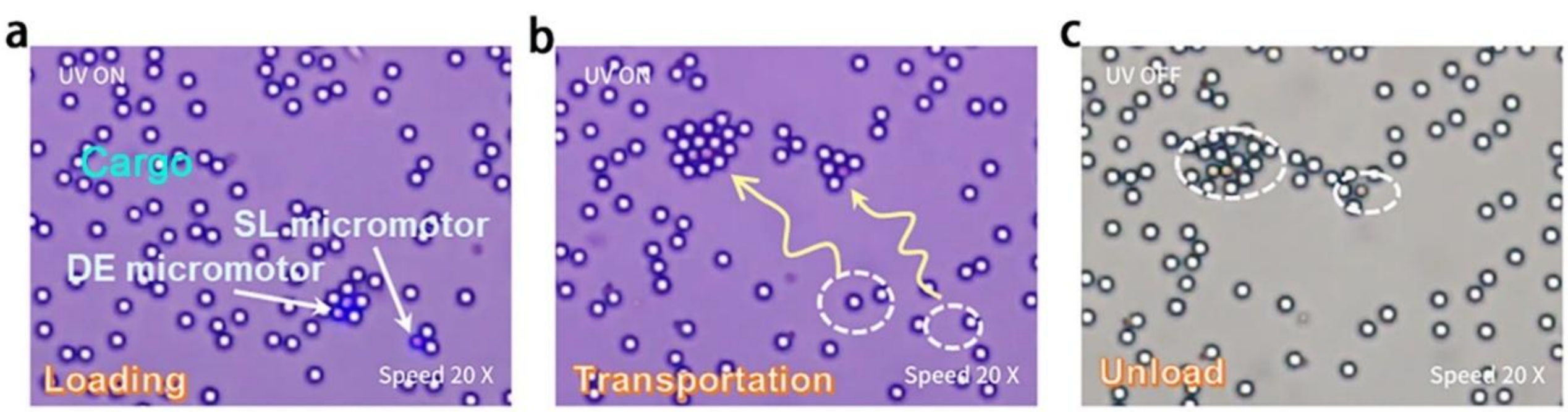

Figure S16. Directional cargo manipulation process of the SL and DE micromotor

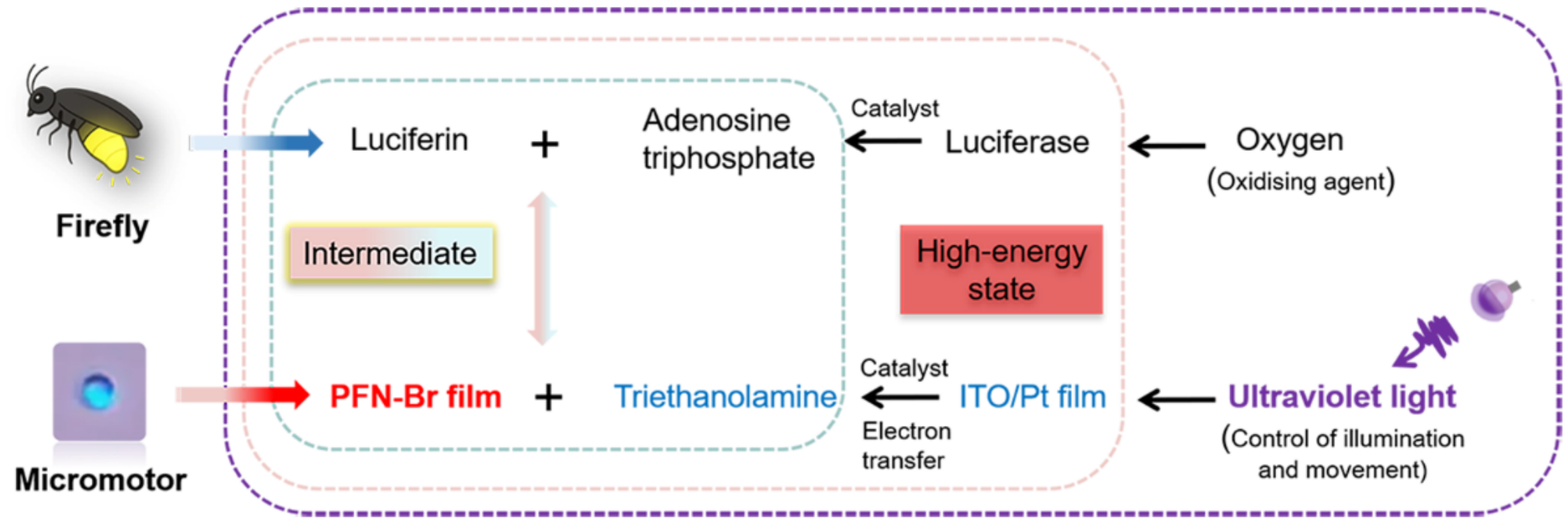

Figure S17. A schematic comparison between the biomimetic luminescence process in SiO_2/Ti/PFN-Br/Pt micro-nano motors and the bioluminescence in fireflies.

Bionic micromotors and the bioluminescence mechanism of fireflies

The phenomenon of bioluminescence in living organisms represents a remarkable example of highly efficient energy conversion occurring at the microscopic scale. Fireflies are particularly illustrative, producing intense visible light through a precisely coordinated biochemical cascade. As shown in Figure 8, luciferin reacts with adenosine triphosphate (ATP) under the catalysis of luciferase to form a high-energy intermediate, which is subsequently 14haracte by molecular oxygen to emit photons. This naturally evolved route—14haracterized by finely regulated electron transfer, enzymatic specificity, and high quantum efficiency—provides a compelling biological model for designing controllable luminescent and self-propelled artificial systems.

In this study, a SiO_2/Ti/ITO/PFN-Br/Pt micromotor is developed to capture the essential features of firefly bioluminescence. The semiconducting polymer PFN-Br, deposited onto the ITO surface, acts as an artificial analogue of luciferin. Under ultraviolet irradiation, PFN-Br absorbs photons and transitions into an excited state, analogous to the high-energy intermediate formed in fireflies. Simultaneously, triethanolamine (TEOA) in the aqueous environment functions as an electron donor, supplying the chemical energy input in a manner comparable to ATP. The ITO/Pt bilayer serves as a synthetic catalytic centre that emulates the role of luciferase by enhancing interfacial electron extraction and transfer. This engineering strategy significantly improves charge separation and enables efficient radiative recombination, giving rise to visible luminescence under UV excitation.

Thus, the artificial system reconstructs key steps of the natural bioluminescence pathway through rational materials design rather than biochemical catalysis.Beyond luminescence, the micromotor also emulates the firefly's concurrent use of biochemical energy for light generation and locomotion. Under UV illumination, the active hemisphere develops a net positive charge, establishing a local electric field that drives motion via self-electrophoretic propulsion. In parallel, the platinum (Pt) layer promotes hydrogen evolution reactions, generating asymmetric ionic and molecular concentration gradients around the motor. This gradient induces additional propulsion through self-diffusiophoresis. The combined contributions of self-electrophoresis and reaction-induced diffusiophoresis give rise to autonomous and directional locomotion. Such coupling between luminescence and motility closely mirrors the dual functionality of fireflies, which simultaneously exploit biochemical pathways for both light emission and movement.

Overall, the SiO_2/Ti/ITO/PFN-Br/Pt micro-nano motor achieves a successful biomimetic translation of the firefly luminescence mechanism into an artificial photo-electrochemical hybrid system. Through the deliberate integration of semiconducting polymers, conductive oxide films, and Pt catalytic layers, the system reconstructs the core functional elements of bioluminescence: (i) photon absorption and formation of excited intermediates, (ii) directional electron transfer and catalytic enhancement, (iii) radiative emission, and (iv) synchronous conversion of energy into mechanical motion. This biomimetic strategy not only deepens our understanding of light–matter interactions at the microscale but also provides a promising pathway toward intelligent micro-nano robotic platforms capable of luminescence, environmental sensing, and programmable actuation.

Table S1. Summary of reported speed-to-length ratios of micromotors driven by ultraviolet or visible light.

	**Light source**	**Body length (μm)**	**Speed (μm/s)**	**Body length/ Speed （bdl/s）**	**Illuminate or not**	**Shape programming**	**Ref.**
**SiO_2/Ti/ITO/PFN-Br/Pt micromotor**	Ultraviolet	5	7.1	1.46	YES	YES	This work
**TiO_2/α-Fe_2O_3 microrobots**	Ultraviolet	2	2.5	1.25	NO	NO	[6]
**Au/B-TiO_2 Micromotors**	Ultraviolet	3.5	2	0.60	NO	NO	[7]
**Si-Au micromotors**	Visible	4	5	1.25	NO	NO	[8]
**GaN/ZnO Microtubes**	Ultraviolet	4.6	5.5	1.19	YES	NO	[9]
**CoO−TiO_2 Microswimmers**	Ultraviolet	6	6.5	1.08	NO	NO	[10]
**Bi_2WO_6 Microrobots**	Visible	7	1.9	0.27	NO	NO	[11]
**$BiVO_4$ Micromotors**	Visible	8	5	0.62	NO	NO	[12]
**Sb_2Se_3/ZnO nanomotor**	Visible	10	12	1.2	NO	NO	[13]
**Si/ TiO_2 micromotors**	Ultraviolet	11	6	0.55	NO	NO	[14]
**TiO_2 microtubes**	Ultraviolet	50	60	1.20	NO	NO	[15]
**TiO_2 plate micromotors**	Ultraviolet	200	24	0.12	NO	NO	[16]

Movie S1

Motion trajectory of the SiO_2/Ti/ITO/PFN-Br/Pt micromotors under illumination at 365 nm with different TEOA concentration.

Movie S2

Motion trajectory of the single SiO_2/Ti/ITO/PFN-Br/Pt micromotor under illumination at 365 nm.

Movie S3

Motion trajectory of the single SiO_2/Ti/ITO/PFN-Br/Pt micromotor under illumination at 365 nm with different UV intensity.

Movie S4

Motion trajectory of the dimers (DE) micromotor under illumination at 365 nm.

Movie S5

Motion trajectory of the two trimer configurations (TR-I and TR-II) micromotor under illumination at 365 nm without magnetic field.

Movie S6

Motion trajectory of SiO_2/Ti/ITO/PFN-Br/Pt/Ni TR-I micromotor under illumination at 365 nm. (Note: The magnetic field is used solely to control the motor's flipping or direction of motion; the magnetic field is removed during operation.)

Movie S7

Motion trajectory of “upright” TR-I micromotor under illumination at 365 nm. (Note: The magnetic field is used solely to control the motor's flipping or direction of motion; the magnetic field is removed during operation.)

Movie S8

The transport capability observer of single and dimers (DE) configuration micromotor under illumination at 365 nm.

Movie S9

Observation of the formation and rotation processes of the DE-RI microrotors under illumination at 365 nm.

Movie S10

Observation of the formation and rotation processes of the DE-RII microrotors under illumination at 365 nm.

Movie S11

The human distress signal SOS and the mating signal behaviour of colony-forming fireflies: the duration of the flash interval corresponds to the length of the Morse code symbol.

References

[1] Z. Hu, X. Zhang, Q. Yin et al., "Highly efficient photocatalytic hydrogen evolution from water-soluble conjugated polyelectrolytes," *Nano Energy* 60, no. (2019): 775, https://doi.org/10.1016/j.nanoen.2019.04.027.

[2] X. Li, H. Shang, Y. Xiong et al., "Silver/jellyfish-like mesoporous polydopamine nanomotor with concentration-dependent synergistic antibacterial activity," *Chemical Engineering Journal* 505, no. (2025): 159367, https://doi.org/10.1016/j.cej.2025.159367.

[3] S. Yang, B. Sun, Y. Liu et al., "Effect of ITO target crystallinity on the properties of sputtering deposited ITO films," *Ceramics International* 46, no. 5 (2020): 6342, https://doi.org/10.1016/j.ceramint.2019.11.110.

[4] S. Mohanapriya, S. Suganthi, V. Raj, "Mesoporous Pt–Ni catalyst and their electro catalytic activity towards methanol oxidation," *Journal of Porous Materials* 24, no. 2 (2016): 355, https://doi.org/10.1007/s10934-016-0268-y.

[5] A. L. Patterson, "The Scherrer Formula for X-Ray Particle Size Determination," *Physical Review* 56, no. 10 (1939): 978, https://doi.org/10.1103/PhysRev.56.978.

[6] M. Urso, M. Ussia, X. Peng et al., "Reconfigurable self-assembly of photocatalytic magnetic microrobots for water purification," *Nature Communications* 14, no. 1 (2023): 6969, https://doi.org/10.1038/s41467-023-42674-9.

[7] B. Jang, A. Hong, H. E. Kang et al., "Multiwavelength Light-Responsive Au/B-TiO_2 Janus Micromotors," *ACS Nano* 11, no. 6 (2017): 6146, https://doi.org/10.1021/acsnano.7b02177.

[8] D. Zhou, Y. C. Li, P. Xu et al., "Visible-light driven Si–Au micromotors in water and organic solvents," *Nanoscale* 9, no. 32 (2017): 11434, https://doi.org/10.1039/c7nr04161f.

[9] N. Wolff, V. Ciobanu, M. Enachi et al., "Advanced Hybrid GaN/ZnO Nanoarchitectured Microtubes for Fluorescent Micromotors Driven by UV Light," *Small* 16, no. 2 (2019): 1905141, https://doi.org/10.1002/smll.201905141.

[10] V. Sridhar, B.-W. Park, S. Guo et al., "Multiwavelength-Steerable Visible-Light-Driven Magnetic CoO–TiO_2 Microswimmers," *ACS Applied Materials & Interfaces* 12, no. 21 (2020): 24149, https://doi.org/10.1021/acsami.0c06100.

[11] K. Villa, L. Děkanovský, J. Plutnar et al., "Swarming of Perovskite-Like Bi_2WO_6 Microrobots Destroy Textile Fibers under Visible Light," *Advanced Functional Materials* 30, no. 51 (2020): 2007073, https://doi.org/10.1002/adfm.202007073.

[12] K. Villa, F. Novotný, J. Zelenka et al., "Visible-Light-Driven Single-Component $BiVO_4$ Micromotors with the Autonomous Ability for Capturing Microorganisms," *ACS Nano* 13, no. 7 (2019): 8135, https://doi.org/10.1021/acsnano.9b03184.

[13] X. Zhan, J. Zheng, Y. Zhao et al., "From Strong Dichroic Nanomotor to Polarotactic Microswimmer," *Advanced Materials* 31, no. 48 (2019): 1903329, https://doi.org/10.1002/adma.201903329.

[14] B. Dai, J. Wang, Z. Xiong et al., "Programmable artificial phototactic microswimmer,"

Nature Nanotechnology 11, no. 12 (2016): 1087, https://doi.org/10.1038/nnano.2016.187.

[15] S. Giudicatti, S. M. Marz, L. Soler et al., "Photoactive rolled-up TiO_2microtubes: fabrication, characterization and applications," *Journal of Materials Chemistry C* 2, no. 29 (2014): 5892, https://doi.org/10.1039/c4tc00796d.

[16] Y. Wang, Z. Li, A. A. Solovev et al., "Light-controlled two-dimensional TiO_2 plate micromotors," *RSC Advances* 9, no. 50 (2019): 29433, https://doi.org/10.1039/c9ra06426e.
