# Supplementary material for: Catalytic Luminescent Polymer Micromotors With Shape‐Programmed Motion
Source: Adv Sci (Weinh). 2026 Oct 22:e78303. Online ahead of print. doi: 10.1002/advs.78303 (PMC13690988; doi:10.1002/advs.78303)
Supplement: Supplementary file 2 [file ADVS-9999-e78303-s006.docx]

**Highlights：**

1. Precise energy-level regulation of the ITO/PFN-Br/Pt layers facilitates high-efficiency photodriven micromotor motion.
2. UV light powers rapid micromotor propulsion, while built-in blue luminescence allows in-situ visual tracking.
3. Shape-programmed trimers micromotor displays highly stable directional motion.
